# All-stage targeted therapy for invasive cryptococcosis through interaction between the secretory protein Cig1 and hemin

**DOI:** 10.1016/j.ajps.2025.101053

**Published:** 2025-03-30

**Authors:** Liting Cheng, Zhongyi Ma, Xinlin Yang, Xue Wang, Yuqiong Wang, Xinlong Liu, Zhongjie Tang, Dingxi Jang, Guojian Liao, Tongbao Liu, Shuang Wu, Chong Li

**Affiliations:** a Medical Research Institute, College of Pharmaceutical Sciences, Southwest University, Chongqing 400715, China; b Guangdong-Hong Kong-Macao Joint Laboratory for New Drug Screening, School of Pharmaceutical Sciences, Southern Medical University, Guangzhou 510515, China

**Keywords:** *Cryptococcus neoformans*, Cig1, Blood-brain barrier, Shared target, Hemin

## Abstract

Cryptococcosis, a serious systemic fungal infection caused by *Cryptococcus neoformans* (*C. neoformans*) and its variants, poses a significant clinical challenge due to its poor prognosis and severe health implications. The treatment of cryptococcal infections is complicated by several unique factors, stemming from both the pathogenic characteristics of the fungi and the biological barriers they exploit. These include the fungi's protective capsule, their ability to reside within host macrophages—thereby evading pharmacological intervention—and their involvement in multi-organ infections such as the lung and brain, in particular their strategic positioning within the brain, protected by the blood-brain barrier (BBB). To overcome these obstacles, precise active targeting emerges as a pivotal strategy. Identifying common targets is imperative to enhance therapeutic efficacy while ensuring the druggability of delivery systems. However, research on the methodology for selecting such shared targets remains sparse. In our investigation, we have pioneered the use of secreted proteins as shared target to trace the pathogens and their infection pathways. We identified the mannoprotein Cig1, prominently expressed on the surfaces of infected macrophages, lungs, and brains, as a viable shared target. On this basis, we utilized Hemin, a ligand for Cig1, to design liposomes (Hemin Lip) tailored for addressing complex fungal infections. By leveraging the interaction with the secreted protein Cig1, Hemin Lip specifically identifies and binds to organs and macrophages harboring cryptococcal infections, thereby facilitating targeted and efficacious clearance of both intracellular and extracellular fungus. Moreover, we have extended this targeting mechanism to other nanomedicinal platforms, including albumin nanoparticles. This study proposes an innovative drug delivery model that targets extracellular secretory proteins within the infection microenvironment, offering a streamlined formulation with the potential for effective therapy against complex infections.

## Graphical abstract

We innovatively proposed a new strategy for drug delivery that utilizes specific extracellular secreted proteins of pathogenic microorganisms as shared targets. We identified the mannoprotein Cig1, notably expressed on the surfaces of infected macrophages, lungs, and brains, as a viable shared target. Building on this, we employed Hemin, a ligand for Cig1, to engineer liposomes (Hemin Lip) tailored for addressing multifaceted fungal infections. By leveraging the interaction with the secreted protein Cig1, Hemin Lip distinctly identifies and binds to organs and macrophages harboring cryptococcal infections, thereby facilitating the targeted and efficacious clearance of both intracellular and extracellular fungal loads.

**Figure:**
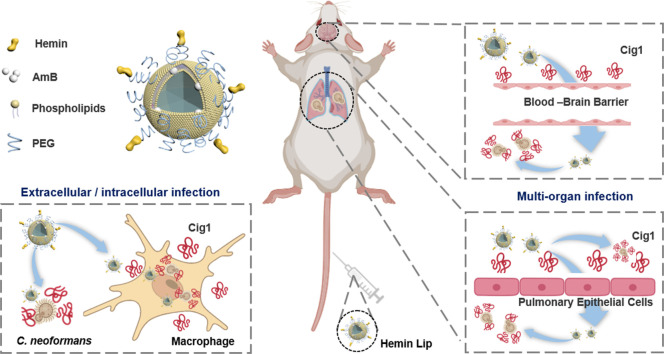

## 1 Introduction

Invasive fungal infections (IFIs) are systemic infections caused by yeasts or molds that invade deep tissues [1]. Clinically, IFIs manifest as systemic diseases affecting internal organs, characterized by organ-level hemorrhage, impaired organ function, and inflammatory exudative lesions. Individuals at the highest risk for IFIs are those with compromised immune systems, such as HIV-positive individuals, cancer patients receiving chemotherapy or immunotherapy, and solid organ transplant recipients[2]. The incidence of IFIs has risen in recent years, resulting in millions of fatalities annually. *C. neoformans*, identified as the leading fungal threat on the World Health Organization's Fungal Priority Pathogens List, is a widespread environmental opportunistic pathogen that causes the deadly disease cryptococcosis [3]. Cryptococcal infection typically initiates in the lungs as cryptococcal pneumonia after inhaling fungal spores or dried yeast cells from the environment, subsequently spreading to the central nervous system (CNS) [4]. This dissemination accounts for ∼70% of related deaths, exceeding 650,000 fatalities annually. Despite the growing need, only a limited number of new antifungal drugs have been approved over the past decade, insufficient to meet clinical demands. Cryptococcal infections are complicated not only by multi-organ involvement but also by several biological barriers that significantly hinder effective treatment. These barriers include (1) an external capsule surrounding the fungal cell wall, which provides additional protection, (2) the ability of *C. neoformans* to evade antimicrobial agents by residing within macrophages, and (3) the impermeability of the blood-brain barrier (BBB) [5] following CNS infection. Therefore, there is a pressing need to develop novel antifungal medications and devise efficient drug delivery strategies that can bypass these obstacles, enhance treatment effectiveness, and reduce toxicity for both current and future antifungal treatments.

Receptor ligand-mediated drug delivery has long been a cornerstone of strategies for active targeted interventions against cells at foci, including various pathogens. Typically, this involves modifying the surface of the drug delivery system (DDS) with ligands that correspond to target proteins on the surface of target cells, thereby facilitating efficient drug delivery through receptor binding. Conceivably, when the disease involves multiple types cells at foci, identifying shared target receptors across various cells is crucial to balance efficacy and druggability. For example, folate receptors are overexpressed in certain breast cancer cells and associated tumor macrophages. This allows for the utilization of folate-modified liposomes to achieve a "kill two birds with one stone" effect, which is more efficient than modifying two separate targeting ligands for cancer cells and macrophages individually [6]. Similarly, the heightened expression of Low-density lipoprotein receptor-related protein-1 (LRP-1) in the BBB, brain gliomas, tumor blood vessel formation, and imitation vessels enables the peptide ligand of LRP-1 to guide polymer micelles for multi-step 'systematic' targeting of brain gliomas [7]. Additionally, the elevated levels of the IL-22 receptor IL-22RA1 in the pancreas and liver have prompted the creation of a short-duration IL-22-bispecific biologic medication that efficiently targets these organs, helping to regulate blood glucose and markedly decrease liver fat accumulation, inflammation, and fibrosis [8]. Although a few studies have discussed about the use of shared targets, there is currently no established method to discover them [9]. This challenge is particularly pronounced in pathogen infections, where the substantial differences in species and structural characteristics between microorganisms and host cells create uncertainty in finding receptor proteins (or homologous proteins) that are specifically expressed on both microorganisms and host cells.

Secreted proteins, including enzymes, antibodies, and some protein hormones, are involved in numerous physiological and pathological processes such as immune regulation and inflammatory responses. The collection of these actively secreted proteins, termed the "secretome", possesses characteristics that make them promising targets for therapeutic intervention [10]. Among these proteins, those that are locally secreted can bind to cell surfaces, making them potential candidates for proteomic studies aimed at biomarker discovery for various diseases [11]. Within the complex milieu of the cellular microenvironment at foci—such as infected organs—these secreted proteins are not only present on the surface of cells but are also prevalent throughout the associated microenvironment. This widespread yet specific presence makes them highly suitable as potential shared targets for overcoming multiple barriers in DDSs. For example, proteins that bind to albumin like SPARC (secreted protein acidic and rich in cysteine) are highly expressed in glioma cells and the tumor microenvironment (TME), including peritumoral stromal cells, aiding the targeted delivery of albumin nanoparticles to brain tumors. Matrix metalloproteinases MMP-2 and MMP-9, also known as gelatinases, are abundantly present in invasive and metastatic tumor cells and the associated TME [12,13]. The C-terminal Tensin-like peptide (CTT) is an inhibitory therapeutic agent that binds specifically to MMP-2 and MMP-9. By utilizing CTT as a homing peptide, liposomes encapsulating a cancer drug can specifically target gelatinase activity sites, thereby inhibiting the growth of human tumor xenografts in mice [14]. Secreted proteins that can serve as shared targets are also found in bacteria. For example, IsdX1 and IsdX2 are two hemophores secreted by Bacillus species to the envelope and extracellular milieu, where they extract heme from hemoglobin and deliver it to the cell wall-bound protein IsdC [15]. Similarly, proteins secreted in specific areas are essential for the development and spread of pathogenic fungi. Based on existing genomic data (https://mycocosm.jgi.doe.gov/), several secretomic databases such as the Fungal Secretome Database [16] and the Fungal Secretome KnowledgeBase [17] have been created, opening more opportunities to identify new shared targets for DDSs. For instance, Csa2, part of the Rbt5 protein group, is released into the surrounding environment and plays a role in extracting iron from human hemoglobin during the hyphal development of *Candida albicans* (*C. albicans*), aiding in the expression of virulence traits [18]. The secretory proteome of Cryptococcus has been systematically studied [19], among which, Cig1 has been particularly associated with virulence and heme uptake, making it a notable candidate for further investigation. These findings indicate that secreted proteins, including Cig1, may be potential targets for therapeutic intervention in fungal infections. Given that the application of secreted proteins as drug delivery targets in tumor-targeted therapies is a really recent development, extending this approach to the treatment of invasive fungal diseases remains a promising and innovative strategy.

In this study, we innovatively confirm that Cig1 is highly expressed on the surface of Cryptococcus, in infected macrophages, as well as in the brains and lungs of infected hosts, suggesting its potential as a shared target for the simultaneous targeting of multiple barriers in cryptococcal therapy. Subsequently, we characterized Hemin as a ligand with a high affinity for Cig1 and used it to prepare Hemin-modified liposomes (Hemin Lip) to combat complex fungal infections. Leveraging the interaction between Hemin and Cig1, we hypothesized that Hemin Lip could effectively recognize and target infected lungs, brains, and macrophages, specifically targeting the *C. neoformans*. These hypotheses were examined using cell cultures and *C. neoformans*-infected mouse models.Notably, we systematically compared the efficacy of liposomes modified with the classic peptide angpep-2, incorporating the antifungal drug amphotericin B (AmB). Furthermore, the use of this innovative targeting approach was investigated in various nanomedicines, including albumin nanoparticles, to evaluate its broader applicability. Our study demonstrates the promising application of Hemin Lip in targeting *C. neoformans*. Utilizing shared targets for all-stage targeting holds promise for enhancing drug targeting efficiency while simultaneously improving druggability, providing a foundation for further development of targeted nanomedicine therapies against fungal infections.

## 2 Materials and methods

### 2.1 Materials

Hemin was acquired from Sigma-Aldrich (St. Louis, Missouri, USA). AmB and coumarin-6 (C6) were obtained from Aladdin Bio-Chem Technology Co., Ltd (Shanghai, China). DSPE-PEG_2k_-NH_2_ and EPC were supplied by A.V.T. Pharmaceutical Co., Ltd (Shanghai, China). A Cy3-labeled AffiniPure goat anti-mouse IgG (H+ L) antibody was obtained from Bioss Biotechnology Co., Ltd (Beijing, China). 1,1′-Dioctadecyl-3,3,3′,3′-tetramethylindotricarbo cyanine iodide (DiR) was purchased from Fluorescence Biotechnology Co., Ltd (Beijing, China). Other chemicals used were of analytical grade.

BEnd.3 cells were obtained from the Pricella Biotechnology Co., Ltd. (Wuhan, China). The cells were grown in Dulbecco's Modified Eagle's Medium (DMEM), procured from KeyGEN, China, and enriched with 10% fetal bovine serum (FBS) sourced from Gibco, USA, along with 80 U/ml penicillin and 0.08 mg/ml streptomycin. The H99 strain of *Cryptococcus neoformans* var. *grubii* (serotype A), obtained from the American Type Culture Collection (ATCC, USA), was cultured in a nutrient-rich yeast extract-peptone-dextrose (YPD) medium.

Female BALB/c mice (6 weeks old, 18–22 g), and Sprague Dawley rats (weighing 180–220 g) were acquired from the SJA Laboratory Animal Co., Ltd, Hunan. These animals were housed in a specific pathogen-free (SPF) facility under a controlled environment, at a temperature of 28 ± 2 °C and a humidity level between 30% and 50%. The animal research facility has received accreditation from the Institutional Animal Care and Use Committee (IACUC) at Southwest University Laboratory Animal Center (IACUC Issue No IACUC-20221114–14). All animal experiments complied with the standards established by the Ethical Review Committee for Experimental Animals at Southwest University in China.

### 2.2 Generation of cig1Δ mutant

The *cig1∆* mutant was created using overlap PCR, following the method described earlier [20]. The 5′ and 3′ segments of each *cig1∆* gene were extracted from H99 genomic DNA. Using M13 primers (M13F and M13R), the primary selectable marker (Neor) was amplified from the pJAF1 plasmid [21]. Overlap PCR with primers was used to create each gene replacement cassette. The purified overlap PCR product was then precipitated onto 10 µl gold microcarrier beads (0.6 µM; Bio-Rad), followed by biolistic transformation of H99 as previously outlined [22]. Stable transformants were chosen on YPD medium with 1 M sorbitol. To identify *cig1∆* gene mutants, diagnostic PCR was performed by analyzing the 5′ junction of the disrupted mutant alleles with specific primers. Positive transformants were identified by PCR screening and subsequently verified by Southern blot analysis.

### 2.3 Synthesis and characterization of Hemin-PEG_2k_-DSPE

The targeting material Hemin-PEG_2k_-DSPE was synthesized through the conjugation of hemin to NH_2_-PEG_2k_-DSPE using an amidation reaction. Initially, NH_2_-PEG_2k_-DSPE (27.3 mg, 4.9 mM) and hemin (19.55 mg, 14.7 mM) were dissolved in 2.0 mL DMF. Following a 15-min reaction, PyBOP (10.6 mg, 4.96 M) was introduced into the mixture while being stirred magnetically. The mixture was left to react for 1 h at ambient atmosphere. Subsequently, the mixture was dialyzed (MWCO 2,000) in deionized water for 2 d to remove any unreacted Hemin and DMF. The mixture was subsequently passed through a 0.22 µm filter to remove insoluble particles, freeze-dried for a full day, and kept at −20 °C. The end product, Hemin-PEG_2k_-DSPE, underwent analysis with a MALDI-TOF mass spectrometer from Bruker Daltonics, Germany. Additionally, after incubating DSPE-PEG_2K_-Hemin in 5% serum at 37 °C for 10 d, the ultraviolet (UV) detection was performed by adding organic solvent to extract DSPE-PEG_2K-_Hemin.

### 2.4 Preparation and characterization of the engineered liposomes

Hemin-modified and AmB-encapsulated liposomes (Hemin Lip/AmB) were produced through the thin-film hydration method. To obtain empty liposomes, a blend of EPC, cholesterol, and NH_2_-PEG_2k_-DSPE (Lip) or EPC, cholesterol, NH_2_-PEG_2k_-DSPE, and Hemin-PEG_2k_-DSPE (Hemin Lip) in a 1:1 chloroform/methanol solution was subjected to rotary evaporation, resulting in a thin lipid film. The lipid film was later moistened with a phosphate-buffered saline solution at 37 °C. The resulting solution was sonicated for 3 min using a bath sonicator operating at 500 W. The lipid dispersion was further filtered using polycarbonate membranes. To prepare liposomes containing C6 or AmB, the same method was used, but C6 or AmB was added prior to forming the thin film (0.05 mg C6 per 5 mg EPC; 0.5 mg AmB per 5 mg EPC). Using a Zetasizer Nano ZS90 from Malvern Instruments, Southborough, MA, the size and zeta potential of the liposomes were determined in deionized water at a lipid concentration of 0.125 mM, after unbound AmB was removed via gel filtration with a Sephadex G-50 column. To determine the drug encapsulation efficiency of the liposomes, AmB was extracted using a DMSO-methanol mixture and analyzed by HPLC at a wavelength of 405 nm.

### 2.5 Preparation and validation of recombinant cig1 proteins and rabbit anti-Cig1 polyclonal antibody

#### 2.5.1 Recombinant cig1 proteins

Recombinant protein expression was conducted using LB bacterial broth. After the culture reached an optical density of 0.6 to 0.8 at a wavelength of 600 nm, IPTG was added at a concentration of 0.5 mM to initiate protein production. The production process then continued at a temperature of 37 °C and a speed of 150 rpm for a duration of 20 h. Following induction, the bacterial culture was centrifuged, subjected to ultrasonication, and centrifuged again. To assess the expression of the recombinant protein, the supernatant and sediment were examined using SDS-PAGE and stained with Coomassie Blue R-250. For purification, the recombinant protein was passed through a Ni-TED Sefinose column and eluted with imidazole at concentrations of 100 mM, 250 mM and 500 mM. Subsequently, the samples were analyzed using through SDS-PAGE followed by staining with Coomassie Blue R-250.

#### 2.5.2 Polyclonal rabbit anti-Cig1 antibodies

Mice were subcutaneously immunized with 100 µg (1 mg/ml) of recombinant Cig1 protein. Tail blood samples were taken before immunization to act as negative control serum. The immunization process was repeated on Day 14 and 28, with blood collection occurring 7 d after each immunization for serum separation. The target antigen (5 µg/ml) was used to coat a 96-well plate, which was then left to incubate at 4 °C for 12 h. Subsequently, this dish was incubated with 5% skim milk under 37 °C for 2 h. Then these wells were treated by the purified antibody, diluted to 1:400, and incubated under 37 °C for 2 h. Following the wash step, a 1:6,000 dilution of goat anti-mouse IgG HRP was introduced, and the plate was kept at 37 °C for 60 min. Ultimately, the optical density (OD) value at 450 nm was recorded.

#### 2.5.3 Western blotting

Total proteins extracted from tissues were analyzed using SDS-PAGE electrophoresis, run at 25 V and 1.3 A for 13 min. The isolated proteins were subsequently transferred to PVDF membranes and blocked using a 5% BSA solution. The membranes were kept at 4 °C for 12 h with a 1:1,000 dilution of mouse anti-Cig1 antibody, then incubated for an additional hour with a 1:10,000 dilution of HRP-conjugated goat anti-mouse IgG (H+L). The protein bands on the PVDF membranes were visualized using a ChemiDoc chemiluminescence gel imaging system (CLINX, Shanghai, China).

### 2.6 Surface plasma resonance (SPR) measurements

The interaction between hemin/DSPE-PEG_2K_-Hemin and Cig1 was measured by SPR (Nicoya Lifescience, Waterloo, Canada). The COOH chip stored at 4 °C was rinsed carefully with PBS buffer to ensure that the surface is free of impurities and absorb excess liquid. In order to attach Cig1 onto the chip, 200 µl NHS (0.02 mol/l) and EDC (0.1 mol/l) in PBS buffer were introduced to activate the carboxyl groups on the chip's surface, with flow rate maintaining 20 µl/min at 25 °C. Cig1 (30 mg/ml) was then suspended in PBS running buffer and injected. To assess the interaction between hemin and Cig1, hemin was initially dissolved in DMSO and subsequently diluted with PBS to various concentrations. The hemin mixtures were introduced, and each concentration was tested in triplicate. After every measurement, the chip's surface was refreshed by 200 µl of 10 mmol/l hydrochloric acid. The SPR instrument used for this measurement was from Nicoya Lifescience (Waterloo, Canada). For analyzing the interaction between Hemin Lip and Cig1, Hemin Lip was immobilized onto a LIP-1 sensor chip. Different concentrations of Cig1 protein were sequentially injected using the same procedure as described above.

### 2.7 Molecular docking

Firstly, the crystal structure of Cig1 protein (Uniport ID: J9VUB8) was predicted using AlphaFold2 [23]. The three-dimensional structure of hemin was acquired from the PUBCHEM database, and the MMFF94 force field was used to minimize energy. Excess chains, ions, and water molecules from the receptor structures were removed using PyMOL software [24]. Subsequently, AutoDockTools software was employed to add hydrogen atoms to the receptor structures and to convert the formats of both ligands and receptors. Molecular docking was then carried out using AutoDock Vina software [25]. The docking results were visualized with PyMOL software.

### 2.8 Preparation and culture of bone marrow‐derived macrophages

Bone marrow‐derived macrophages (BMDMs) were obtained from the tibia and femur bones of female C57BL/6 (8 weeks) mice, using a protocol outlined in a recent study [26]. The bones with their muscles and connective tissues taken off, were superficially sterilized with 75% ethanol. Then the ends of the bones were cut off, and bone marrow was collected by rinsing the femur and tibia with 2 ml DMEM containing 5% fetal bovine serum (FBS). Bone marrow from one mouse was gathered, centrifuged at 1,000 rpm for 5 min. The cells were rinsed and suspended in a solution containing DMEM with 20% FBS, 1% streptomycin/penicillin (10,000 U/ml), and 20 ng/ml murine macrophage/monocyte-colony stimulating factor (MCSF; FineTestProteins). Cells from 2 mouse hind legs were cultured in a 24-well plate (Corning) under 5% CO_2_ at 37 °C. The fluid was changed on Day 3 and 5, and could be used for follow-up experiments on Day 7.

### 2.9 In vitro interaction of Hemin Lip with infected BMDMs

To analyze interactions *in vitro*, BMDMs were used in this experiment at suitable concentrations (1 × 10^7^ cells/ml). *C. neoformans* and the *cig1∆* mutant in logarithmic phase were harvested, rinsed three with PBS, then treated with 15 % mouse complement for 60 min in a cell incubator at 37 °C. Afterward, 1 million cryptococcal cells were introduced into each well and left to incubate for 1 d. This well was rinsed four times with PBS to eliminate extracellular cryptococcal cells. Subsequently, each well received 2.5 µg/ml C6-tagged Lip and was left to incubate for 2 h. Observations were made using a fluorescence microscope (Leica, Germany) to capture images.

### 2.10 In vitro BBB transcytosis assay

To create a monolayer cell culture, bEnd.3 endothelial cells from mouse brains were placed in the upper compartment of Transwell inserts (Corning, USA). The monolayer was deemed ready for use once the transendothelial electrical resistance exceeded 300 Ω. C6-labeled liposome (Lip) was added to the upper chamber and incubated for 4 h. Following incubation, the bEnd.3 cells were washed three times with PBS. Subsequently, pictures were taken with an Operetta CLS high-content screening imaging system (HCS, PerkinElmer). For the infected BBB microenvironment model, the upper layer consisted of a monolayer of bEnd.3 cells representing the BBB, while the lower compartment contained infected BMDMs. The uptake of C6-labeled liposomes in this system was assessed using the same procedure as described above.

### 2.11 Immunofluorescence of Cig1 in the infected BMDMs

As outlined earlier, BMDM cells underwent infection and were subsequently fixed in 4% paraformaldehyde for 0.5 h. The cells were treated with 5% bovine serum albumin (BSA) and left to incubate overnight at 4 °C with an anti-Cig1 antibody. The second day, they were incubated with a Cy3-labeled secondary antibody for 60 min at ambient temperature, then stained with DAPI. A fluorescence microscope (Leica, Germany) was utilized for imaging analysis.

### 2.12 Distribution of Hemin-Lip in living organisms and isolated tissues

#### 2.12.1 Establishment of C. neoformans infection model

The *cig1*Δ mutant strain cells *or C. neoformans* wild-type H99 strain cells, which were intended for infection, were grown in YPD broth overnight at 30 °C. Afterwards, the medium was taken away, and these cells were rinsed four times with PBS, then diluted to a concentration of 2 × 10^6^ CFU/ml. To develop the infection model, a 50 µl dose of the *cig1*Δ mutant strain cells or *C. neoformans* suspension was administered to BALB/c mice via intranasal delivery.

#### 2.12.2 Pharmacokinetic study

A study on pharmacokinetics was performed with female SD rats weighing between 180 and 220 g. The rats were randomly assigned to two groups and intravenously injected with Lip/AmB and Hemin Lip/AmB at an amphotericin B (AmB) dose of 2 mg/kg body weight. Blood samples of 500 µl were drawn from the retinal vein at various intervals: 15 min, 30 min and 1, 2, 4, 8, 12 and 24 h after injection. The sample was centrifuged at 10,000 rpm for 10 min to separate the plasma. Before analysis, AmB was extracted from the plasma by adding 0.8 ml methanol to 0.2 ml the plasma sample. The solution was agitated for five minutes using a vortex mixer, followed by centrifugation at 12,000 rpm for 10 min. The resulting supernatant was collected and analyzed by HPLC.

#### 2.12.3 In vivo and ex vivo imaging of Hemin Lip

To track *in vivo* and *ex vivo* in real-time, Lip and Hemin Lip were tagged with DiR marker. Following a 48-h infection phase, both *C. neoformans*-infected and healthy mice received injections of the labeled compounds through the tail vein. An *in vivo* imaging system (PerkinElmer, USA and Carestream, USA) was utilized for live imaging at designated intervals (1, 4, 8, 12 and 24 h). Mice were sacrificed 24 h post-administration for *ex vivo* imaging. The primary organs (including the heart, brain, spleen, liver, lungs, and kidneys) were gathered, washed with cold PBS, and examined using the imaging system.

#### 2.12.4 Fluorescence co-localization of Hemin Lip/C6 and C. neoformans

Confocal laser scanning microscopy (CLSM) was used to evaluate the specific targeting of Hemin Lip to infected sites in lung and brain. At the beginning, the mice models received tail injections of C6-labeled Lip and Hemin Lip, with a C6 dosage at 0.2 mg/kg. The mice were euthanized after 4 h, and their lung and brain tissues were meticulously removed, embedded in OCT compound, frozen, and sliced into 20 µm thick sections. The samples were treated with 4% paraformaldehyde for 0.5 h, then blocked using 5% BSA, and left to incubate overnight at 4 °C with anti-Cig1 antibody. The following day, the segments were treated with a Cy3-linked secondary antibody for 60 min at ambient temperature, then stained with DAPI. The fluorescence signals were then observed using CLSM.

#### 2.12.5 Tissue distribution studies

To investigate the tissue distribution of Hemin Lip/AmB, infected mice were randomly assigned to two groups, receiving either Lip/AmB or Hemin Lip/AmB at a single dose of 2 mg/kg AmB via tail vein injection. The mice were euthanized at 1, 2, 4 or 8 h post-administration. Primary tissues were gathered, washed with cold PBS, and weighed carefully. Each tissue sample was then mixed and homogenized with 1 ml methanol. The mixtures were spun at 12,000 rpm for 10 min to isolate the supernatant, which was then examined using HPLC. Three mice were used for each time point to ensure adequate data collection.

### 2.13 In vitro anti-fungal activity

For MIC evaluation, suspensions of wild-type H99 *C. neoformans* (10^6^ cells/ml) in fresh YPD medium were dispensed into 96-well plates at 100 µl per well. Afterwards, free AmB, Lip/AmB, A2 Lip/AmB, and Hemin Lip/AmB were introduced to each well in concentrations spanning from 0.125 µg/ml to 16 µg/ml. The dishes were kept at 30 °C for 2 d Cell growth was assessed by visible turbidity. The minimum inhibitory concentration (MIC) of AmB was identified as the smallest amount where no visible growth occurred.

### 2.14 In vivo therapeutic effect

Survival study: The infected mice were divided into four groups at random after 1 d of infection, and received a single intravenous treatment of either saline, Lip/AmB, A2 Lip/AmB or Hemin Lip/AmB, with an AmB dosage of 2 mg/kg. The survival rates of each group were observed over a 32-d period following the treatment.

### 2.15 In vivo toxicity study of hemin lip

The healthy mice were randomly assigned to four groups and received tail vein injections of either saline, Lip/AmB, A2 Lip/AmB or Hemin Lip/AmB, each at a dose of 2 mg/kg AmB. Whole blood was collected 24 h post-injection, and plasma was separated for biochemical analysis. Two kidney function markers (UREA-urea nitrogen and CREA-creatinine) and Liver markers (AST-aspartate aminotransferase, ALT-alanine aminotransferase) were assessed with an automated biochemical analyzer from Shandong BIOBASE Co., Ltd., Jinan, China. Key organs such as the heart, brain, lungs, spleen, liver, and kidneys were preserved in a 4% paraformaldehyde solution. Slices of tissue were prepared and dyed using hematoxylin and eosin (H&E) stain. Subsequently, these segments were analyzed for histopathology using a light microscope (Leica, DMi8, GER).

### 2.16 Non-invasive assessment of GFR

Transcutaneous kidney filtration rate (GFR) measurement was conducted following similar procedures as previously reported [27]. The BALB/c mice were anesthetized using isoflurane, and their dorsal fur was removed. An imaging device (Mannheim Pharma and Diagnostics GmbH, Mannheim, Germany) was meticulously attached to the shaved backs of the mice with double-sided tape and a bandage. During the test, anesthesia was suspended, and the animals were allowed to remain conscious in a single cage. A baseline signal was captured for 5 min, and the FITC-labeled inulin was injected intravenously (50 mg/kg). The animals were then kept in an undisturbed dark environment, and the signal was recorded for an additional 1.5 h. After the recording period, the chip was removed, and the data were automatically analyzed using MPD Studio software ver.RC6 (MediBeacon GmbH, Cubex41, Mannheim, Germany).

### 2.17 Preparetion of Hemin@BSA complex

The Hemin@BSA complex was prepared through a straightforward combination of BSA and hemin. Specifically, 1.775 mg BSA was dissolved in 5 ml PBS to create solution A (final concentration: 0.356 mg/ml). Separately, 6.3 mg hemin powder was dissolved in 1 ml DMSO, resulting in solution B with a concentration of 6.3 mg/ml. Subsequently, 5 ml solution A was mixed with 37.5 µl solution B, followed by incubation at 37 °C for 15 min. After incubation, the mixture was dialyzed (MWCO 1,000) in deionized water for 24 h to remove DMSO, and stored at 4 °C. To optimize the co-assembly conditions, varying volumes of solution B (5 to 50 µl) were combined with solution A and incubated under identical conditions for further study.

### 2.18 Statistical analysis

Statistical analyses were conducted using GraphPad Prism 8 software. The data are presented as the mean ± standard deviation (SD) and were analyzed using two-tailed unpaired Student's *t*-test, one-way ANOVA, two-way ANOVA and survival curves. Differences were considered statistically significant at *P* < 0.05. *P<0.05, **P < 0.01, ***P < 0.001 and ****P < 0.0001.

## 3 Results and discussion

### 3.1 Identification of high expression of Cig1 in all-stage of C. neoformans infection

**Fig. 1:**
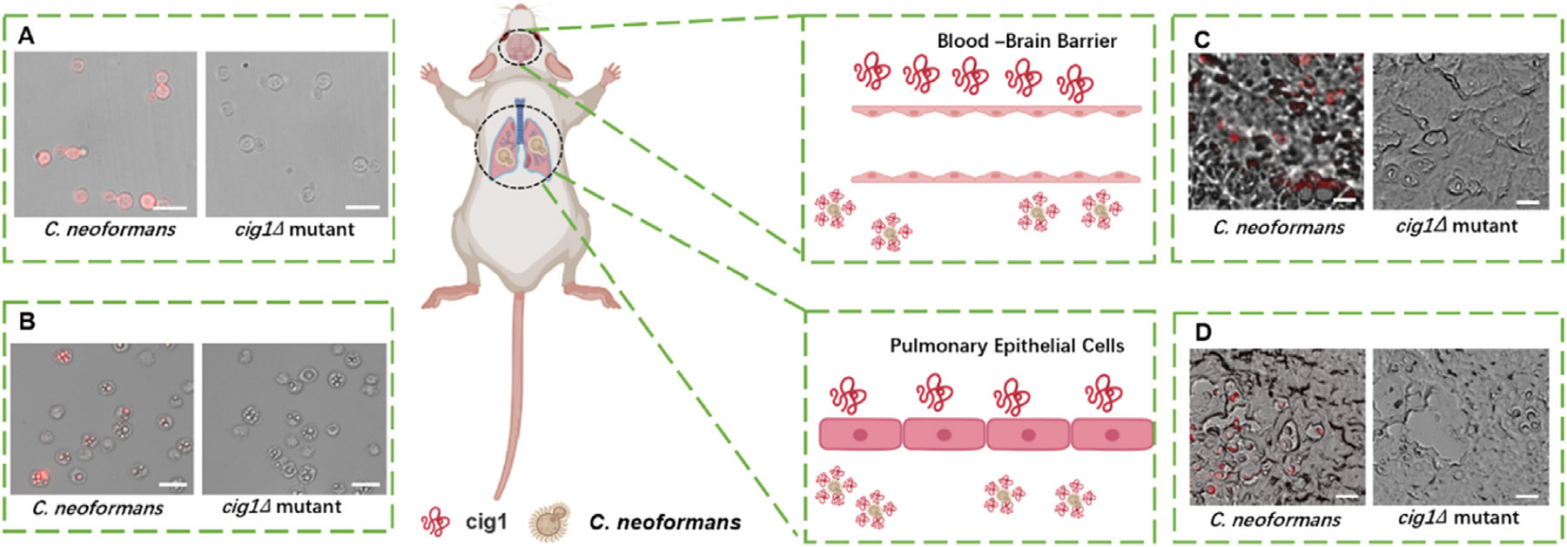
Identification of Cig1 high expression in *C. neoformans* and multiple stages of infection. (A) Cig1 expression in *C. neoformans* H99 and *cig1∆* mutant (Scale bar: 10 µm). (B) Cig1 expression in *C. neoformans* H99 and *cig1∆* mutant infected BMDMs. (C) Cig1 expression in *C. neoformans* H99 and *cig1∆* mutant infected brains. (D) Cig1 expression in *C. neoformans* H99 and *cig1∆* mutant infected lungs. (Scale bar of B-D: 20 µm).

The progression of invasive cryptococcal infections involves several stages, each characterized by different biological barriers that must be overcome. When delivering drugs across numerous target cells, it is crucial to consider that each cell type may express different proteins. This necessitates the use of multiple ligands, potentially complicating the drug delivery system. Locally secreted proteins that bind to cell surfaces offer a novel approach to simplify DDS by using shared targets. In the pathogenic fungus *C. neoformans*, Cig1 proteins have been demonstrated in the literature to act as extracellular secreted proteins that localize to the fungal cell walls and capsules. However, it has not been established whether Cig1 proteins are distributed in the microenvironment of cryptococcal infections. Therefore, we investigated and demonstrated that the extracellular secretory protein Cig1 is widely distributed at the infection foci, making it a potential shared target at multiple points in the cryptococcal disease process. Specifically, our study revealed that Cig1 is distributed not only on the surface of *C. neoformans* (Fig. 1A, left) but also on the surface of macrophages (Fig. 1B, left) and on the vascular endothelium of the brain (Fig. 1C, left) and lung (Fig. 1D, left), all of which were infected by *C. neoformans*. In contrast, in the *cig1∆* mutant, there was no Cig1 distribution observed on *C. neoformans* (Fig. 1A, right), macrophages (Fig. 1B, right), brain (Fig. 1C, right), or lung vascular endothelium (Fig. 1D, right) post-infection. These findings suggest that a delivery system targeting the Cig1 protein is expected to function at various stages of the cryptococcal infection disease course, thus facilitating a more streamlined and effective targeted DDS.

### 3.2 Preparation and characterization of Hemin Lip/AmB

**Fig. 2:**
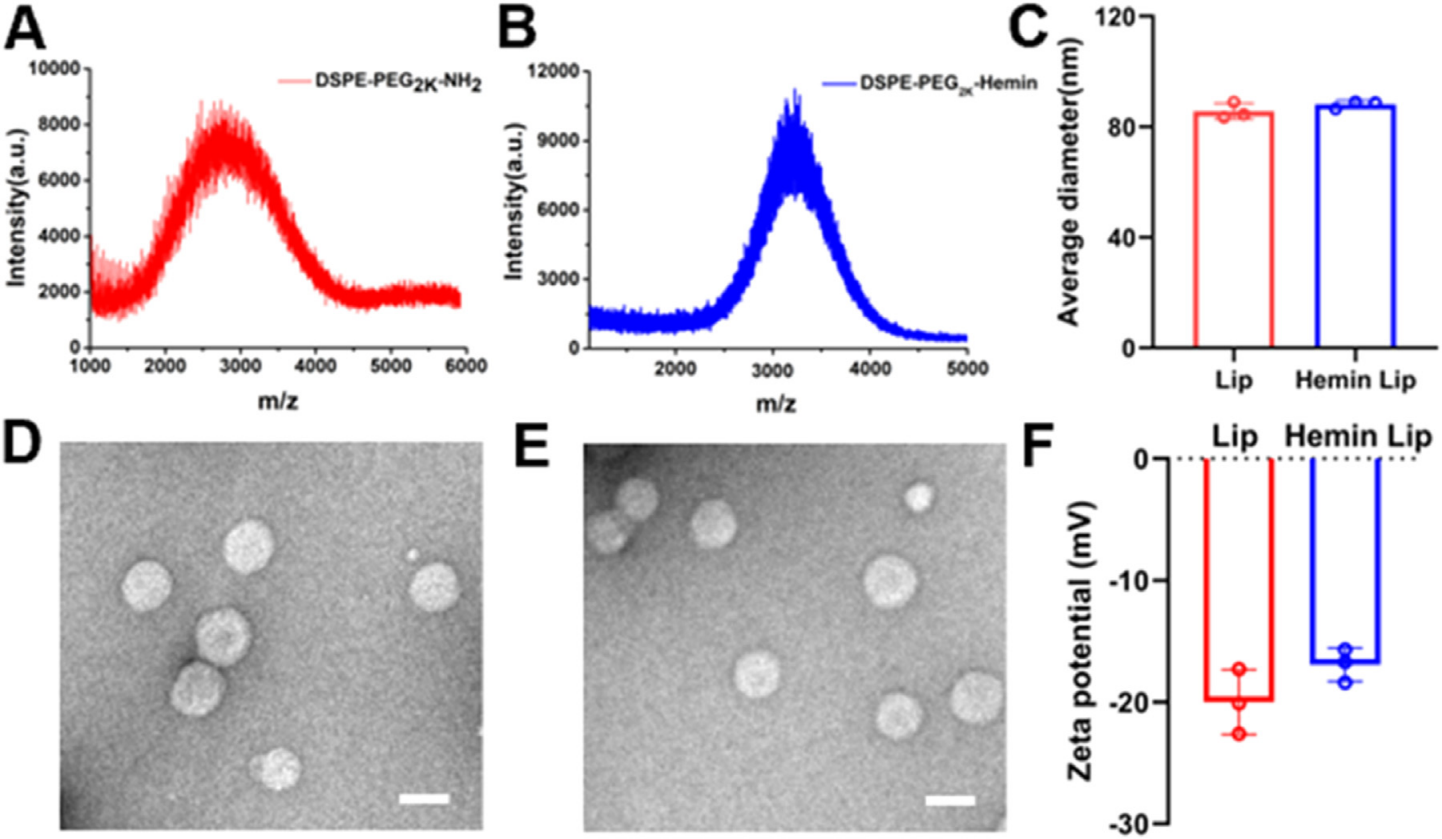
Preparation and Characterization of Hemin Lip/AmB. The MALDI-TOF-MS analysis of DSPE-PEG_2K_-NH_2_ (A) and DSPE-PEG_2K_-Hemin (B). (C) The Hydrodynamic size of Hemin Lip/AmB and Lip/AmB (*n* = 3). TEM images of (D) Hemin Lip/AmB and (E) Lip/AmB (Scale bar: 100 nm). (F) The zeta potential of Hemin Lip/AmB and Lip/AmB (*n* = 3).

To support their growth requirements, many pathogenic fungi have evolved heme transport systems that allow them to obtain heme from the host as a primary source of iron ions [28]. The aforementioned shared target, mannoprotein Cig1, functions to support *C. neoformans* in obtaining iron from host heme. Therefore, using heme as a targeting ligand is anticipated to facilitate targeted delivery by exploiting the mediating role of the shared target Cig1 [29]. In our study, the targeting compound was synthesized by linking hemin to DSPE-PEG_2k_-NH_2_ via a dehydration condensation reaction. The MALDI-TOF-MS assay indicated a rightward shift in the peak after the conjugation of hemin to DSPE-PEG_2k_-NH_2_ (Fig. 2A and 2B). The molecular weight (MW) peak of DSPE-PEG_2k_-NH_2_ was observed at ∼2600, while that of DSPE-PEG_2k_-Hemin appeared at ∼3250. This shift indicates the successful conjugation of hemin, which has a MW of roughly 650, to DSPE-PEG_2k_-NH_2_. The stability of DSPE-PEG_2k_-Hemin was further evaluated. When incubated in 5% serum at 37 °C for 10 d, its UV absorption profile remained unchanged (Fig. S1), indicating that the conjugated linker maintained its integrity under physiological conditions and could be suitable for drug delivery applications.

Hemin Lip/AmB were prepared using a thin-film hydration method [30]. The size of Hemin Lip/AmB was 85.6 ± 2 nm with a narrow polydispersity index (PDI) of <0.2 (Fig. 2C). The particle size remained stable at 4 °C over a 72-h period, demonstrating good physical stability (Fig. S2). The UV absorption spectra of DSPE-PEG_2k_-Hemin and Hemin Lip showed characteristic absorption peaks of Hemin, with a prominent peak at 385 nm (Fig. S3). Transmission electron microscopy (TEM) imaging confirmed that the liposomes were spherical and uniform in size (Fig. 2D and 2E). The liposomes that were developed have a rather high negative ζ-potential, measuring roughly −20 mV. This indicates that the liposomes have a high level of stability, as seen in Fig. 2F. Furthermore, encapsulation efficiencies were determined to be 89.2% for Lip/AmB and 93.4% for Hemin Lip/AmB (Fig. S4), with drug loading efficiencies of 8.9% and 9.3%, respectively (Fig. S5). Additionally, at 48 h, approximately 60% of cumulative release of AmB was observed for both Lip/AmB and Hemin Lip/AmB, indicating consistent sustained release behavior between the two formulations (Fig. S6). In conclusion, DSPE-PEG_2k_-Hemin was successfully synthesized and modified onto liposomes. Based on the size, ζ-potential, and encapsulation efficiency, the prepared liposomes represent promising drug delivery carriers.

### 3.3 Evaluation of the Hemin Lip in vitro

**Fig. 3:**
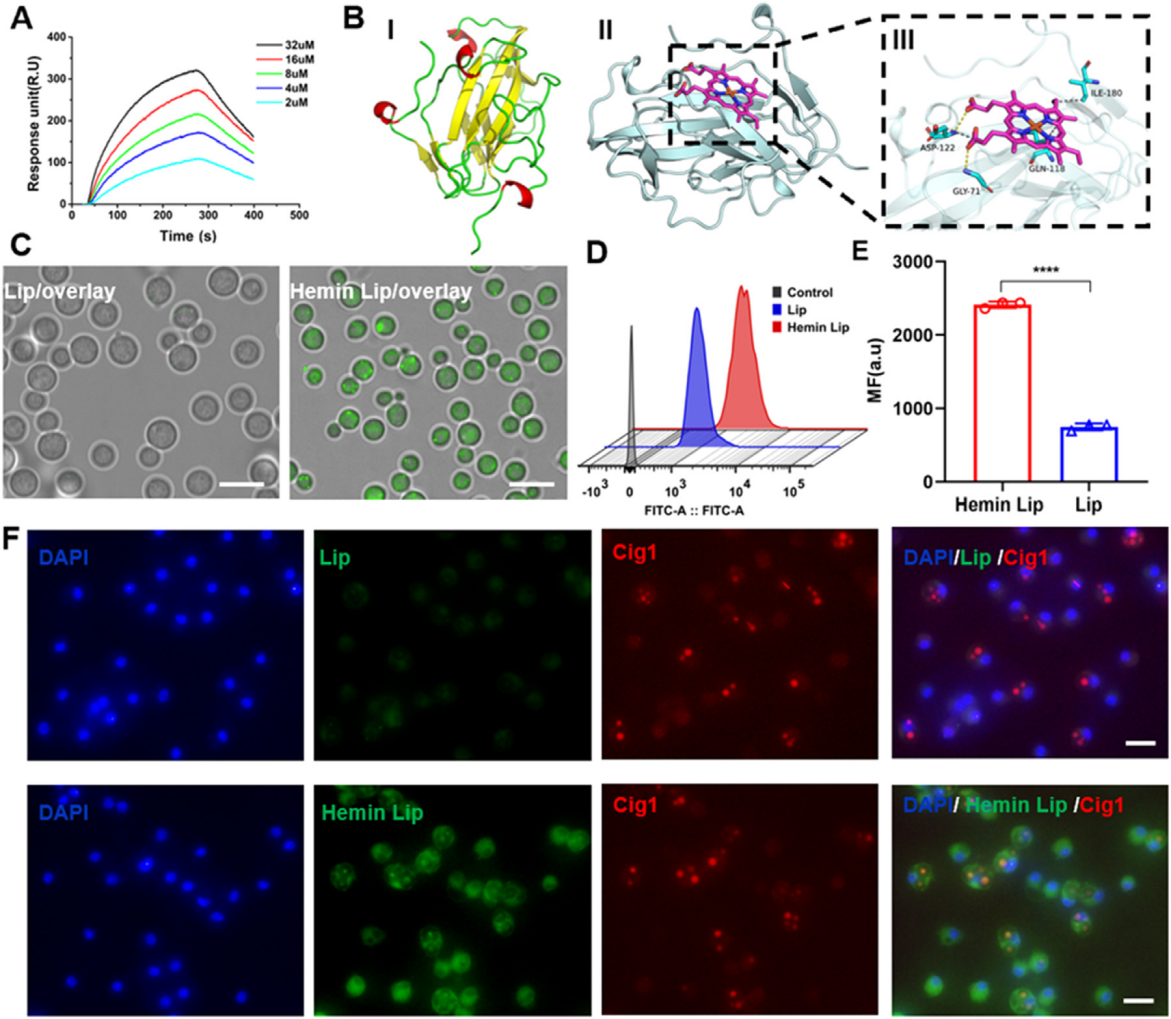
Evaluation of the Hemin Lip *in vitro*. (*A) SPR* response units showing the interaction between hemin and Cig1. (B) 3D ligand-protein interaction pattern of hemin with the Cig1 binding site. I: Predicted crystal structure of Cig1 (UniProt ID: J9VUB8). II: Overall view. III: Local view, where the small molecule is represented as a magenta stick, the protein as a cyan cartoon, hydrogen bonds as yellow lines, and hydrophobic interactions as gray dotted lines. (C) Fluorescent images of Hemin Lip uptake by *C. neoformans* H99 (Scale bar: 10 µm). (D) Flow cytometry analysis of *C. neoformans* H99 after treatment with Hemin Lip and Lip for 2 h. (E) Quantification of the mean fluorescence intensities (MFI) in (D). (F) CLSM images of *C. neoformans* H99 infected BMDMs (Scale bar: 20 µm).

To quantitatively assess the affinity between Cig1 and hemin, we utilized SPR. As shown in Fig. 3A, Cig1 specifically interacted with hemin, exhibiting a binding constant (Kd) of 9.81×10^‒7^ mol/l, which is significantly stronger than that of pheophytin A (Fig. S7), a compound with a similar chemical structure to hemin. Additionally, DSPE-PEG_2k_-Hemin and Hemin Lip demonstrated notable binding affinity for Cig1, with a binding constant (Kd) of 3.2 × 10^–6^ mol/l and 1.2 × 10^–6^ mol/l (Fig. S8), respectively. Further molecular docking analysis indicated that hemin favorably interacts with Cig1 (Fig. 3B). The docking binding energy between Cig1 and hemin was −8.2 kcal/mol, indicating a strong affinity. Hemin formed hydrogen bonds with GLY-71 and ASP-122 in the ligand-binding domain of Cig1, and strong hydrophobic and van der Waals interactions were observed with residues including GLN-118, ILE-180 and ASP-122.These results collectively suggest that hemin has a high affinity for Cig1.

To determine the optimal ratio of DSPE-PEG_2k_-NH_2_ to DSPE-PEG_2k_-Hemin, we studied the targeting capability of Hemin Lip in *C. neoformans* H99. Fluorescence analysis using C6-labeled Hemin Lip indicated that the 2% Hemin Lip treatment group exhibited significantly higher fluorescence in *C. neoformans* H99 compared to other groups, suggesting the efficacy of hemin as a targeting ligand against *C. neoformans* (Figs. S9 and S10). Earlier research indicates that *cig1*Δ mutants demonstrate heightened resistance to harmful hemin analogs relative to wild-type strains, suggesting that the Cig1 protein is crucial for heme acquisition by *C. neoformans* [31]. Therefore, we constructed Cig1 knockout mutants for further investigation (Fig. S11). Our findings revealed that Hemin Lip showed markedly stronger fluorescence compared to Lip in *C. neoformans* H99 (Fig. 3C and S12). Quantitative fluorescence-activated cell sorting (FACS) analysis showed a similar trend (Fig. 3D and 3E). There was no significant uptake of Hemin Lip or Lip in the *cig1*Δ mutant, indicating that Cig1 is crucial for the specific delivery of Hemin Lip (Fig. S13 and S14).

Considering that *C. neoformans* can survive phagocytosis, proliferate within, and escape from phagolysosomes [32,33], we also examined the ability of Hemin Lip to target *C. neoformans*-infected macrophages and subsequently target intracapsular pathogens after entry into macrophages. F4/80 and CD11b are classic macrophage markers. After BMDM isolation, their purity was determined to be approximately 98.0% using flow cytometry with these markers (Fig. S15). BMDMs were then infected with either *C. neoformans* H99 or the *cig1*Δ mutant, followed by incubation with C6-labeled Lip. An anti-Cig1 antibody was successfully produced for subsequent experiments. In Hemin Lip-treated H99-infected cells, a significant overlap between Cig1 and C6 signals was observed (Fig. 3F), whereas no notable uptake was detected in *cig1*Δ-infected cells treated with Hemin Lip or Lip alone (Fig. S16). Moreover, Hemin Lip uptake was markedly higher in infected BMDMs compared to uninfected ones, indicating a specific targeting mechanism for infected cells (Fig. S17). These results indicate that Cig1, located on the surface of *C. neoformans*, can be readily accessed by Hemin-modified Lip. This interaction could potentially improve the ability of drug delivery systems to target specific areas in complex circumstances.

### 3.4 In vivo targeting of Hemin Lip

**Fig. 4:**
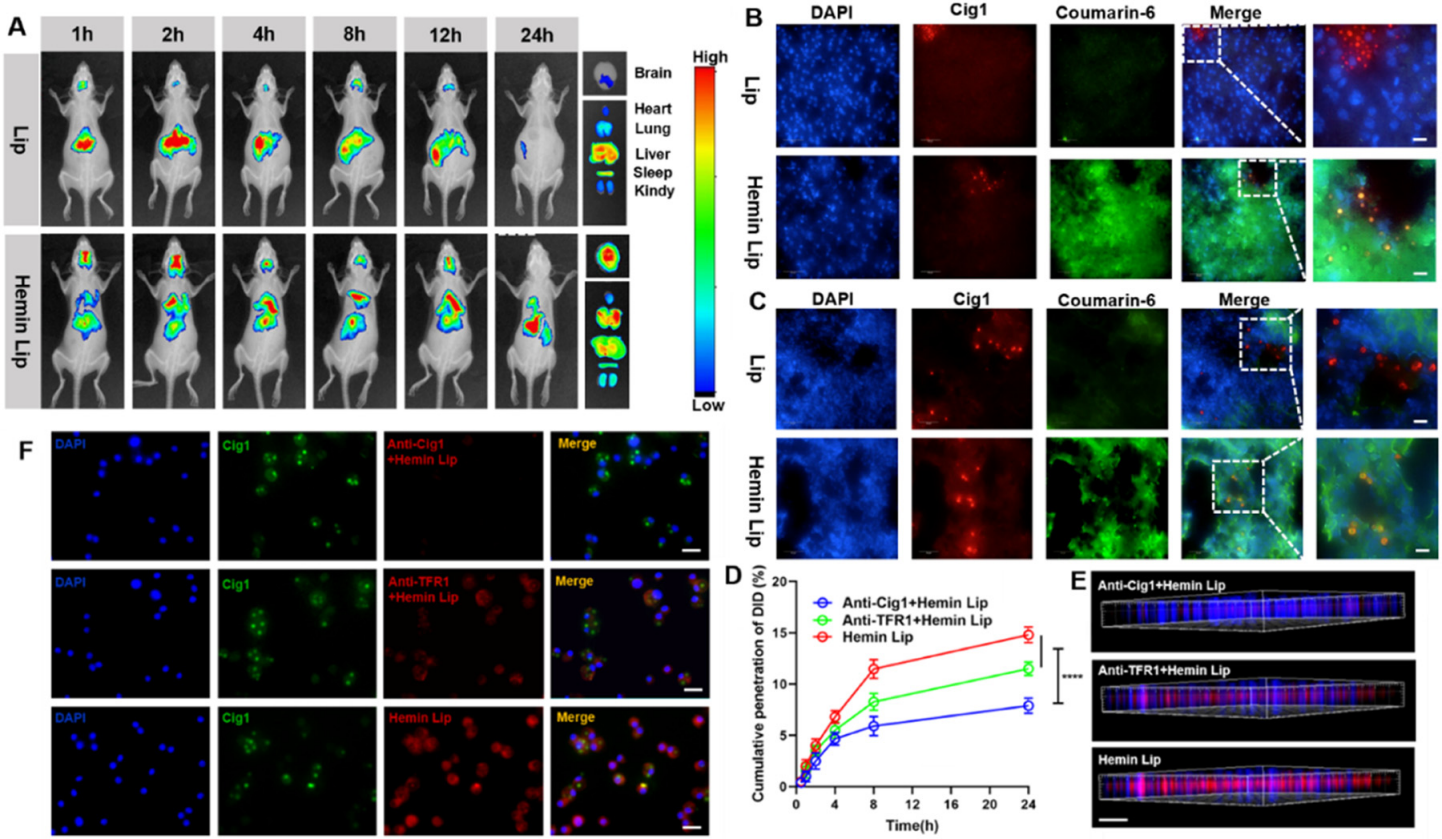
*In vivo* targeting of Hemin Lip. (A)The targeting of Hemin Lip and Lip in infected mouse *in vivo* (*n* = 3). (B) Brain sections from *C. neoformans* H99-infected mice after administration of Hemin Lip and Lip (Scale bar: 10 µm). (C) Lung sections from *C. neoformans* H99-infected mice after administration of Hemin Lip and Lip (Scale bar: 10 µm). *In vitro* permeation of Hemin Lip across bEnd.3/*C. neoformans* H99 cocultured cell monolayers and subsequent targeting of *C. neoformans* H99-infected BMDMs. (D) Cumulative penetration of DiD ( %). (E) Three-dimensional microscopic images of cell monolayers interacting with Hemin Lip (Scale bar: 1000 µm). (F) CLSM images of *C. neoformans* H99 infected BMDMs. (Scale bar: 20 µm).

To assess the *in vivo* targeting ability of Hemin Lip, Lip/DiR, and different proportions of Hemin Lip/DiR, they were administered via intravenous injection into the tail veins of healthy mice. Compared to Lip/DiR and other formulations, the 2% Hemin Lip/DiR exhibited specific accumulation in the brains of healthy mice (Fig. S18 and S19). Additionally, the 2% Hemin Lip demonstrated substantial penetration in an *in vitro* BBB model compared to other groups (Fig. S20). To further investigate the uptake mechanism of Hemin Lip in bEnd.3 cells, these cells were treated with various inhibitors, including chlorpromazine, cyclodextrin, colchicine, and brefeldin A. A significant reduction in Hemin Lip uptake was observed following pre-incubation with chlorpromazine, suggesting that Hemin Lip internalization into bEnd.3 cells occurs via a clathrin-mediated endocytic pathway (Fig. S21). Previous studies have reported significant expression of transferrin receptor 1 (TFR1) on the surface of bEnd.3 cells [34], which exhibits a strong affinity for heme [35]. When anti-TFR1 antibodies were applied to inhibit TFR1, a reduction in Hemin Lip uptake was observed in bEnd.3 cells (Fig. S22). These results indicate that Hemin Lip interacts with TFR1 on bEnd.3 cells and crosses the BBB via clathrin-mediated transcytosis pathways. Subsequently, a mouse model of *C. neoformans* was established using the nasal drop method. The imaging of Hemin Lip in the *C. neoformans* infection mice model showed significant accumulation in the lungs and brains compared to Lip (Fig. 4A, S23 and S24). To better measure targeting effectiveness and biodistribution, Hemin Liposomes containing AmB (Hemin Lip/AmB) and Liposomes with AmB (Lip/AmB) were administered via intravenous injection into the tail veins of infected BALB/c mice, followed by dissection of major organs at 1, 4, 8 and 12 h after injection. As shown in Fig. S25, at each time point examined, Hemin Lip/AmB showed a markedly greater distribution of AmB in the brain and lungs than Lip/AmB. The pharmacokinetic profiles of Lip and 2% Hemin Lip were similar in rats (Fig. S26 and Table S1).

Hemin Lip demonstrated both good lung and brain targeting, effectively recognizing *C. neoformans*. CLSM images of *C. neoformans* H99*-*infected lung and brain sections revealed significantly higher pulmonary and brain accumulation of Hemin Lip compared to Lip (Fig. 4B and 4C). By isolating alveolar macrophages from both healthy and infected mice, we observed that Cig1 was specifically expressed on alveolar macrophages from infected mice. Immunohistochemical analysis further corroborated that Cig1 expression was prominently localized in the lungs and brain, while no significant expression was detected in other major organs (Fig. S27 and S28). The strong colocalization of Cig1 and C6 fluorescence signals in frozen lung and brain sections demonstrated that C6-labeled Hemin Lip effectively crossed the BBB and specifically targeted *C. neoformans in vivo*. In contrast, no significant red or green fluorescence was observed in lung and brain sections infected with the *cig1*Δ mutant (Fig. S29 and S30), highlighting the importance of the Cig1-mediated uptake mechanism. Furthermore, in a coculture system comprising bEnd.3 monolayers and *C. neoformans*-infected BMDMs, we observed that Hemin Lip was able to traverse the BBB and further target infected BMDM cells. When anti-Cig1 antibodies were added, the ability of Hemin Lip to penetrate the BBB and target infected BMDMs was markedly reduced. In contrast, adding an anti-TFR1 antibody did not overly affect its ability to cross the BBB and infect BMDMs. (Fig. 4D–4F). These results demonstrate that Hemin Lip predominantly traverses the BBB and targets infected BMDMs through its interaction with Cig1. These findings suggest that Hemin Lip possesses strong *in vivo* targeting capabilities, effectively penetrating the lungs and brain to recognize and accumulate in *C. neoformans*-infected tissues, thus demonstrating the potential for enhanced drug delivery in complex infection environments.

### 3.5 Therapeutic efficacy of Hemin Lip/AmB

**Fig. 5:**
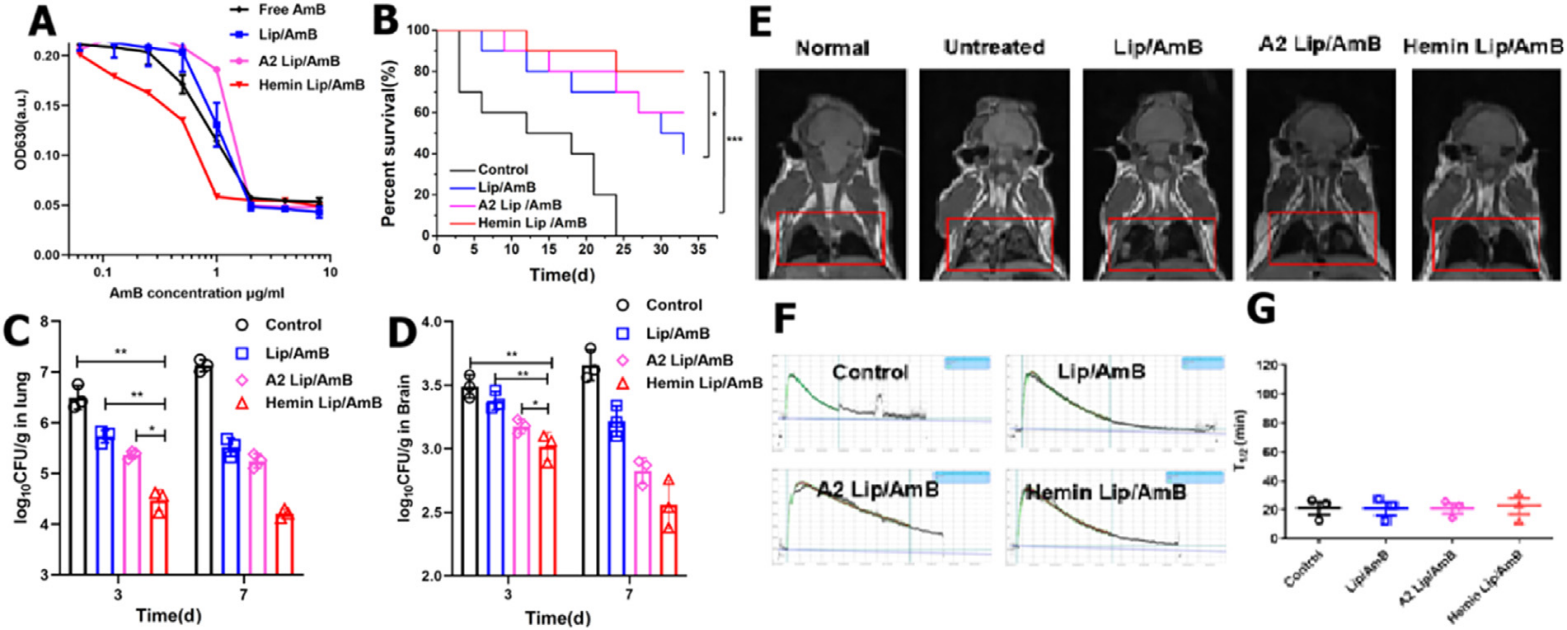
The efficacy of Hemin Lip/AmB. (A) Antifungal experiments of different AmB preparations *in vitro* (*n* = 3). (B) Survival rate of infected mice after treatment with different formulations (*n* = 10). (C) CFU counts in the lungs of the infected mouse after treatment with different formulations (*n* = 3, *P < 0.05, ^⁎⁎^*P* < 0.01). (D) CFU counts in the brains of infected mice after treatment with different formulations (*n* = 3, *P < 0.05, ^⁎⁎^*P* < 0.01). (E) MRI evaluation of infection signals *in vivo* after different treatments (*n* = 3). (F) Observation of nephrotoxicity in mice after administration of different preparations by small animal real-time monitoring system and (G) T_1/2_ of the renal fluorescence elimination. (*n* = 3).

AmB is known for its strong antifungal efficacy, but it is also associated with infusion-related reactions and nephrotoxicity. By altering its biodistribution, liposomal amphotericin B exhibits reduced toxicity and has been commercially available since the 20th century. In this study, hemin was further modified into the existing AmB liposome preparation to enhance therapeutic efficacy. Angiopep-2 is known for its strong affinity for LRP-1, which is extensively expressed on endothelial cells of the brain and lung microvasculature [36,37]. Consequently, Angiopep-2 modified liposome (A2 Lip) served as the control group to evaluate the effectiveness against Hemin Lip/AmB. Based on the *in vitro* antifungal results presented in Fig. 5A, it can be found that Hemin Lip/AmB demonstrated higher effectiveness compared to free AmB, Lip/AmB and A2 Lip/AmB. The growth of *C. neoformans* was significantly inhibited by the Hemin Lip/AmB group at a dose of 1 µg/ml AmB. Preliminary studies evaluating the therapeutic effects of Hemin-modified liposomes at various concentrations revealed that liposomes with 2% Hemin modification provided optimal therapeutic outcomes in both lung and brain tissues (Fig. S31). Consequently, subsequent experiments utilized this specific concentration. For the *in vivo* efficacy study, three groups of *C. neoformans*-infected mice were administered 2 mg/kg of AmB-loaded preparations, and the remaining group was given saline as a control. Over the 32-d observation period, Hemin Lip/AmB was found to significantly prolong survival time (Fig. 5B). In the absence of treatment, all mice died within 24 d, but 80% of the mice treated with Hemin Lip/AmB survived. Moreover, the total number of colony-forming units (CFU) in the lungs and brains of the experimental animals exhibited a significant decrease after 3 and 7 d of Hemin Lip/AmB therapy compared to other groups (Fig. 5C and 5D). Magnetic resonance imaging (MRI) was used to observe pulmonary infection signals *in vivo*. MRI signals were minimal (appearing black) in healthy mice, whereas untreated infected mice displayed elevated MRI signals (appearing white), indicating a severe fungal infection. The Hemin Lip/AmB group achieved full recovery without any signs of infection, outperforming the other groups (Fig. 5E). Microscopic analysis showed significant presence of *C. neoformans* in the pulmonary and cerebral tissues of untreated mice. Conversely, mice that received Hemin Lip/AmB treatment exhibited evident indications of improvement in their lung and brain tissues (Fig. S32 and S33). Furthermore, macrophage extracts revealed that intracellular cryptococci were nearly entirely eliminated following the application of Hemin Lip/AmB (Fig. S34). Additionally, Hemin Lip/AmB at 1 mg/kg, when combined with the clinical drug 5-fluorouracil (5-FC), exhibited therapeutic effects comparable to those of Hemin Lip/AmB at 2 mg/kg alone (Fig. S35&S36 and Table S2). These results suggest that, apart from monotherapy, the combination of Hemin Lip/AmB and 5-FC could present a promising strategy for enhancing antifungal therapy while reducing side effects in complex infection scenarios.

In addition, the toxicity of the liposomal formulation was also assessed *in vivo*. Key physiological indicators and H&E staining outcomes showed that AmB treatment was well-tolerated by mice within the Hemin Lip/AmB formulation (Fig. S37 and S38). The delivery systems, primarily consisting of liposomes, showed good degradability and biocompatibility *in vivo*. Due to the serious nephrotoxicity associated with AmB, the inulin clearance rate was used to evaluate the mice's kidney function 24 h after treatment. Hemin Lip/AmB showed no significant kidney toxicity relative to healthy mice (Fig. 5F and 5G). The findings suggest that Hemin Lip/AmB is exceptionally biocompatible and safe, rendering it appropriate for targeted drug delivery in the treatment of intricate *C. neoformans* infections.

**Fig. 6:**
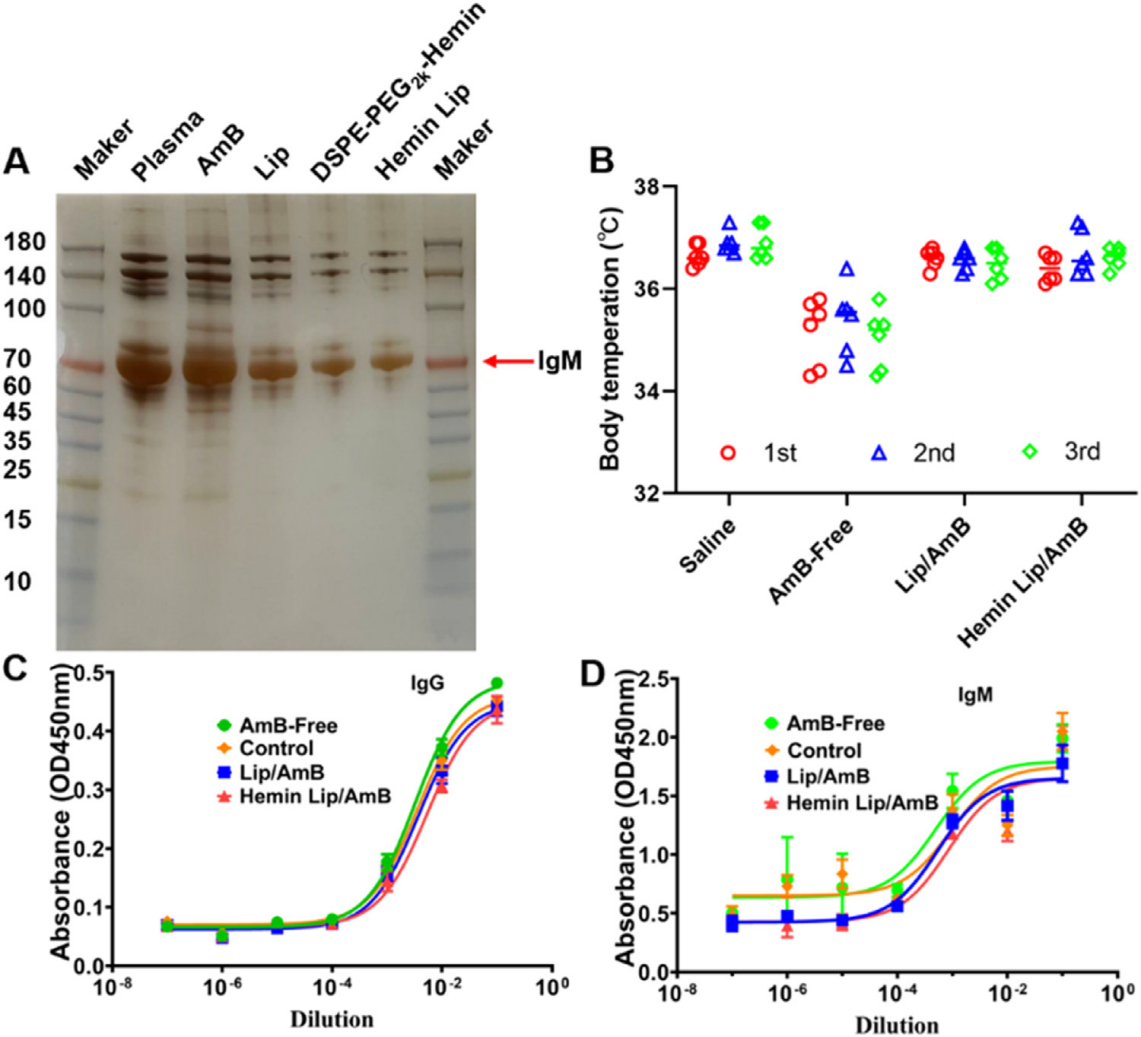
Evaluation of the immunocompatibility of Hemin Lip. (A) Protein corona separated by SDS-PAGE and stained with silver. (B) Body temperature measured 0.5 h post-injection with saline and different liposomes (*n* = 6). ELISA analysis of (C) IgG and (D) IgM levels in serum after injection of different liposomes (*n* = 3).

Recent studies have shown that modifications to conventional positively charged peptide ligands on PEGylated liposomes can increase IgM adsorption, thereby negatively impacting their immunocompatibility [38,39]. To assess the immunogenicity of Hemin Lip, the protein corona formed on its surface was analyzed using SDS-PAGE. As illustrated in Fig. 6A, both Lip and Hemin Lip adsorbed fewer proteins, both in quantity and diversity, compared to AmB-Free. To further evaluate the safety profile, body temperature variations were monitored in mice following repeated administrations. The Hemin Lip group maintained stable body temperatures without fluctuations, suggesting a negligible risk of acute allergic reactions or hypothermia (Fig. 6B). Additionally, serum levels of IgM and IgG were quantified using ELISA (Fig. 6C and 6D), and no significant elevation in immune complex levels was observed, further demonstrating favorable immunocompatibility. Cytokine concentrations, including IL-8, IL-10 and TNF-α, were also measured post-administration. Hemin Lip did not induce excessive secretion of these cytokines (Fig. S39), thereby preventing heightened immune responses that could be harmful to the host. Collectively, these results highlight that Hemin Lip minimizes protein adsorption, maintains a low immunogenic profile, and exhibits advantageous immunocompatibility.

### 3.6 Hemin modification presents a novel targeting platform

**Fig. 7:**
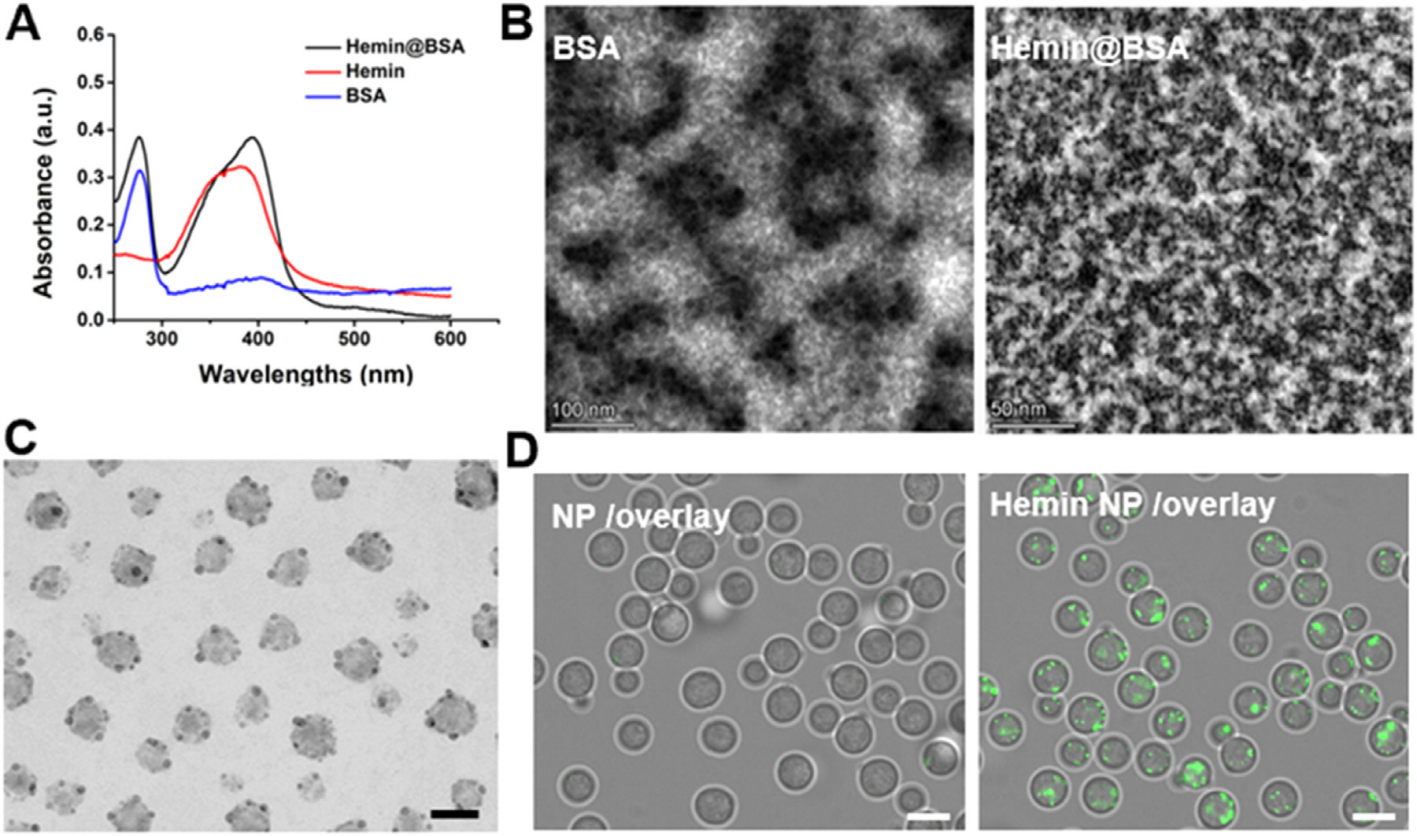
Preparation and characterization of Hemin@BSA NP (A) Comparison between the UV–vis absorption spectra of BSA, hemin and Hemin@BSA. (B) TEM images of BSA scaffolds and the Hemin@BSA composite. (C) TEM images of Hemin@BSA NP (Scale bar: 200 nm). (D) Fluorescent images of BSA NP and Hemin@BSA NP uptake by *C. neoformans* H99 (Scale bar: 5 µm).

In the aforementioned experiments, hemin was successfully modified on liposomes, and Hemin Lip was verified to target *C. neoformans*. In order to investigate whether heme could serve as a versatile target ligand for modifying various nanocarriers or formulations. To explore this idea further, we prepared Hemin@BSA by modifying BSA with Hemin. According to Fig. 7A, the UV–vis spectra of Hemin@BSA complex were compared with BSA and native Hemin. The Hemin@BSA composite showed two absorption peaks at 288 and 405 nm, which match the absorption peaks of BSA (280 nm) and hemin (385 nm). In contrast to native hemin, Hemin@BSA exhibited a red shift in its absorption peak, suggesting an alteration in hemin's conformational structure following its interaction with BSA. Unlike the original BSA segments (Fig. 7B), the Hemin@BSA composites were evenly distributed, exhibiting flake-like structures with an approximate diameter of 50 nm. Hemin@BSA was then prepared into nanoparticles (Hemin@BSA NP, Fig. 7C), with BSA nanoparticles used as a control. The TEM images of BSA NP and the average sizes of BSA NP and Hemin@BSA NP are shown in Fig. S40 and S41. As expected, Hemin@BSA NP also demonstrated the ability to target *C. neoformans* (Fig. 7D). These findings indicate that modified Hemin could be a flexible foundation for developing specialized drug delivery systems. Furthermore, due to the widespread presence of heme transport systems in pathogenic microorganisms, heme-modified nanocarriers hold significant potential for broad application in the treatment of complex pathogenic microbial infections.

## 4 Conclusion

In summary, we have innovatively proposed a new strategy for drug delivery that leverages specific extracellular secreted proteins of pathogenic microorganisms as shared targets. In the context of complex invasive cryptococcal infections, we identified Cig1, an extracellular heme carrier protein secreted by *C. neoformans*, as a shared target. Subsequently, we demonstrated that hemin can be used as a high-affinity ligand, leading to the development of an active targeted liposome DDS capable of effectively overcoming multiple barriers and targeting both intra- and extracellular infections. Finally, we validated the generalizability of this strategy across different nano-formulations. Given that heme transport system is prevalent in various pathogens, this strategy promises to provide novel and effective pathways for anti-pathogen therapy, enabling “simple” formulations to be effective against complex infections.

## Conflicts of interest

The authors declare no competing interest.

## Appendix Supplementary materials

Image, application 1
