# Supplementary material for: All-stage targeted therapy for invasive cryptococcosis through interaction between the secretory protein Cig1 and hemin
Source: Asian J Pharm Sci. 2025 Mar 30;20(4):101053. doi: 10.1016/j.ajps.2025.101053 (PMC12329525; doi:10.1016/j.ajps.2025.101053)
Supplement: Supplementary file 1 [file mmc1.docx]

**Supporting Information**

**All-stage targeted therapy for invasive cryptococcosis through interaction between the secretory protein Cig1 and hemin**

Liting Cheng^a,b,1^, Zhongyi Ma^a,b,1^, Xinlin Yang^a^, Xue Wang^a^, Yuqiong Wang^a^, Xinlong Liu^a^, Zhongjie Tang^a^, Dingxi Jang^a^, Guojian Liao^a^, Tongbao Liu^a^, Shuang Wu^a,*^, Chong Li^a,b,*^


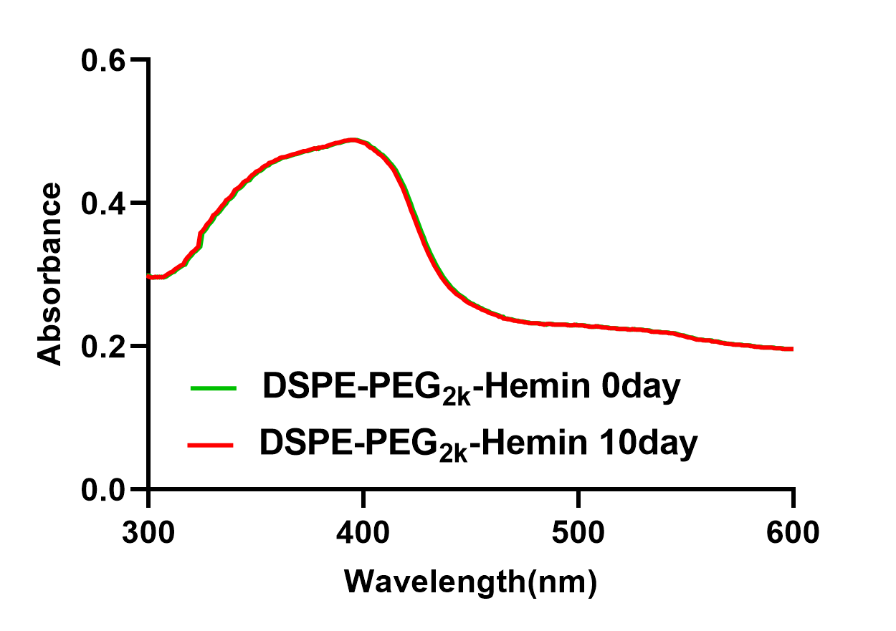


**Fig.S1.** The UV absorption spectra of DSPE-PEG_2K_-Hemin stored at 37°C in a 5% serum solution for 10 days.


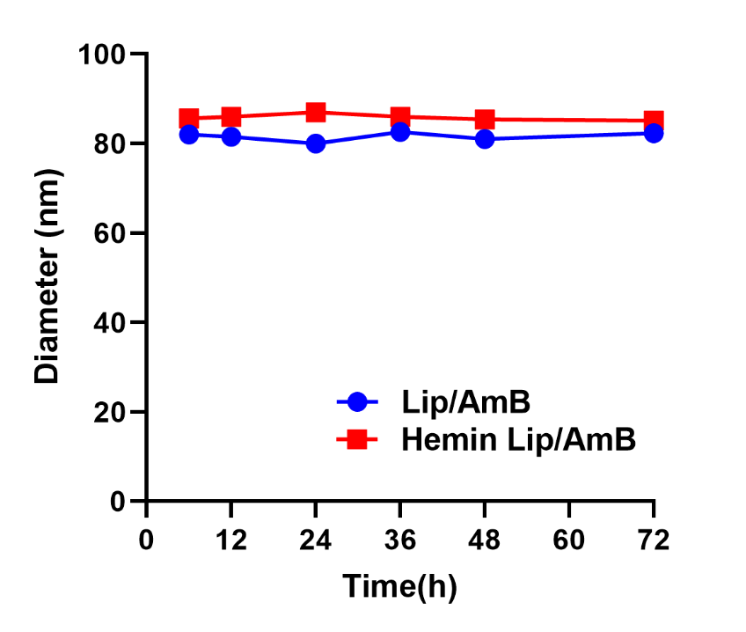


**Fig.S2.** Stability test results of Lip/ AmB and Hemin Lip/ AmB.


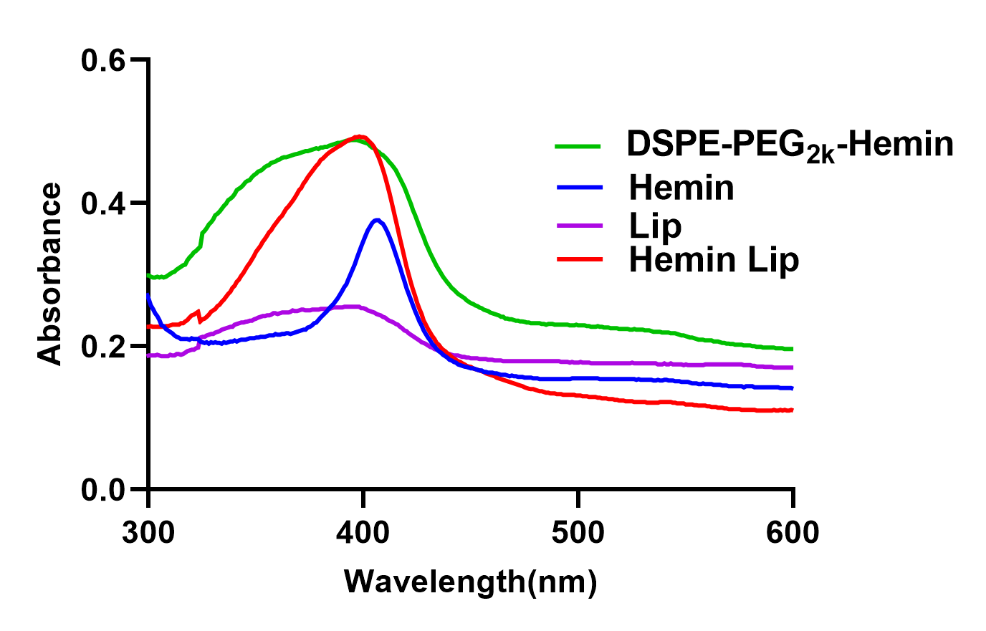


**Fig.S3.** UV absorption spectra of DSPE-PEG_2K_-Hemin, Hemin, Lip, and Hemin Lip.


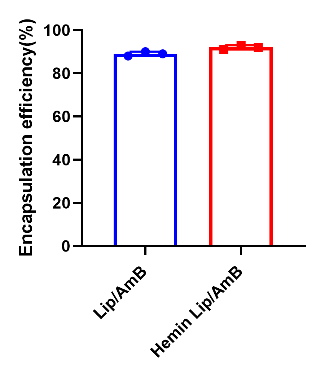


**Fig.S4.** The encapsulation efficiency of Lip/AmB and Hemin Lip/AmB. Data are presented as mean ± SD (n = 3).


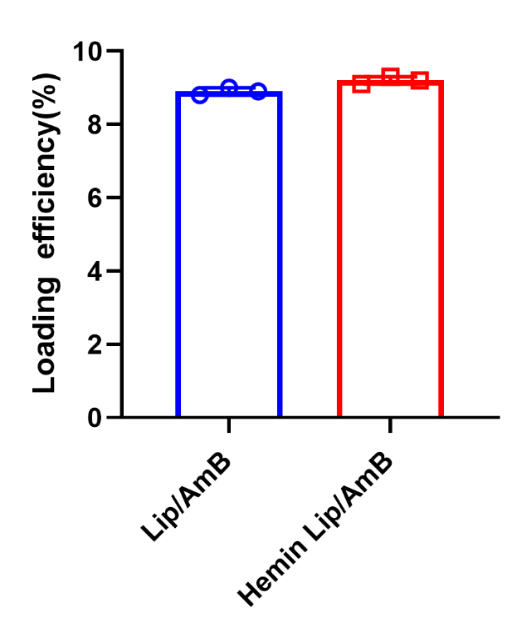


**Fig.S5.** AmB loading efficiency of Lip/AmB and Hemin Lip/AmB. Data are presented as mean ± SD (n = 3).


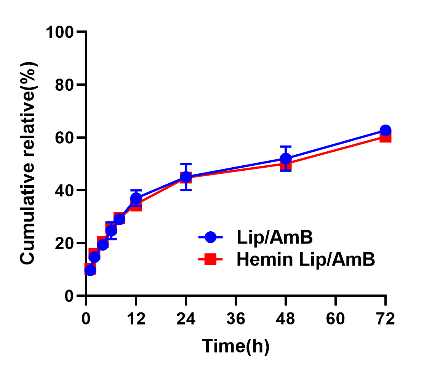


**Fig.S6.** *In vitro* release of AmB from different preparations. The release condition was PBS (pH 7.4) with 2% SDS at 37 °C. Hemin Lip exhibited similar release rates to Lip. Data are presented as mean ± SD (n = 3).

**Fig.S7.** (A) Chemical structure of hemin. (B) SPR response analysis of the affi-nity between the Cig1 and pheophytin A, using a pheophytin A concentration


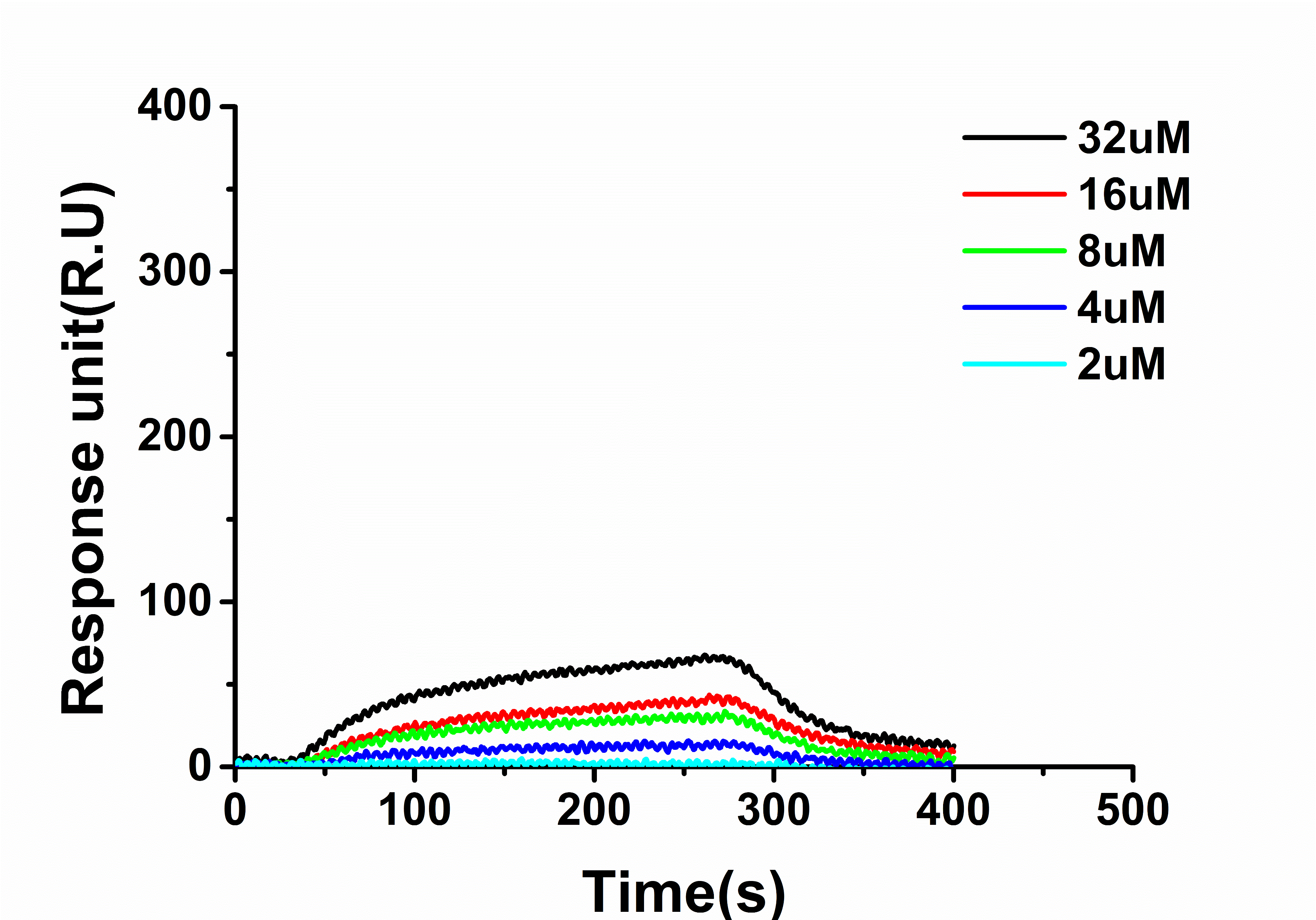

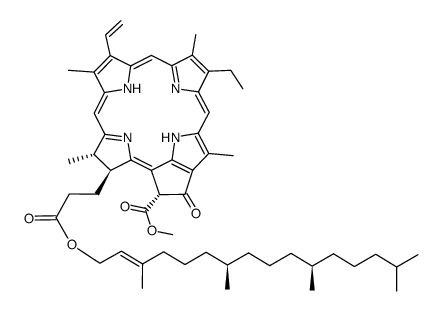

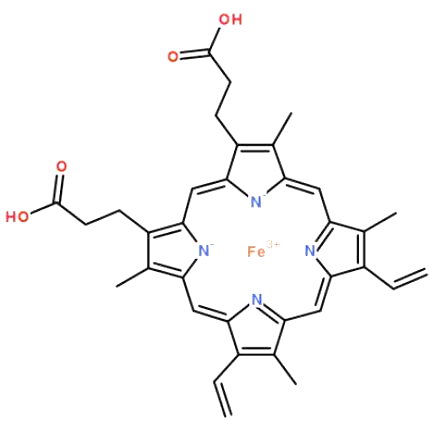


**A**

**B**

range of 2–32µM.

**
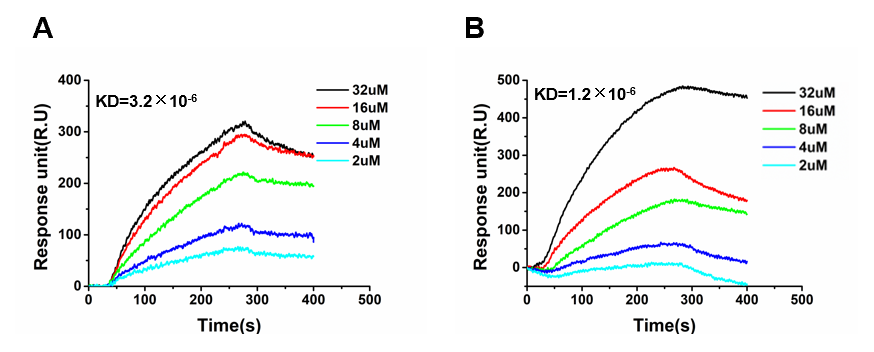
**

**Fig.S8.** (A) SPR response analysis of the affinity between Cig1 and DSPE-PEG_2K_-Hemin, using a DSPE-PEG_2K_-Hemin concentration range of 2–32 µM. (B) SPR response analysis of the affinity between the Hemin Lip and Cig1,

using a Cig1 concentration range of 2–32 µM.


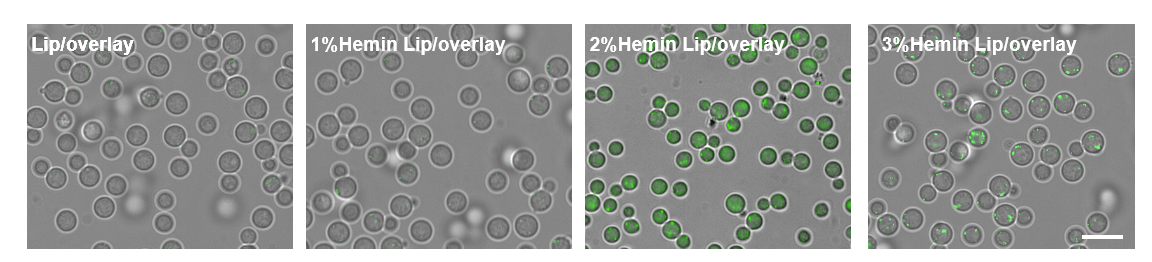


**Fig.S9.** Screening the optimal ratio of DSPE-PEG_2k_-NH_2_/DSPE-PEG_2k_-

Hemin. When the ratio of DSPE-PEG_2k_-Hemin was 2%, the targeting capability in *C. neoformans* H99 was significantly higher than that of other groups.

(Scale bar, 10 μm).


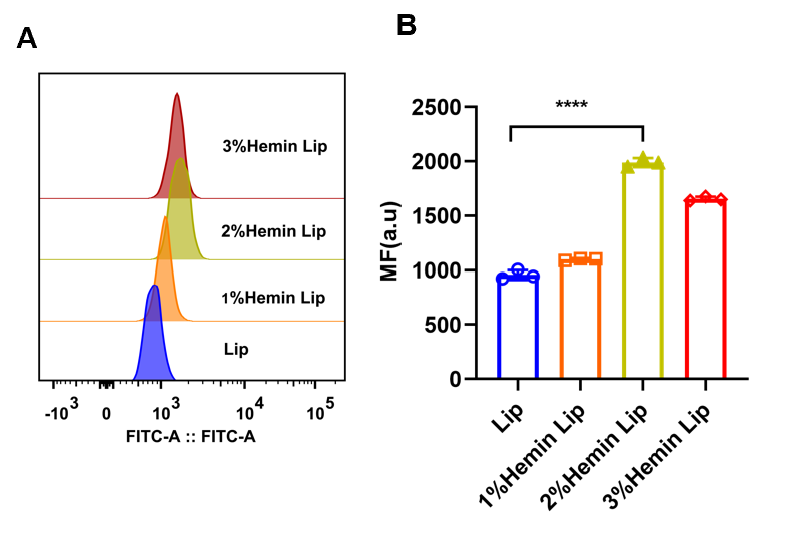


**Fig.S10.** *C. neoformans* H99 uptake of Lip and Hemin Lip (with different Hemin modification ratios) by flow cytometry (A) and the mean fluorescence

intensity of Coumarin-6 was determined by flow cytometry assay (B). Data are presented as mean ± SD (n = 3).


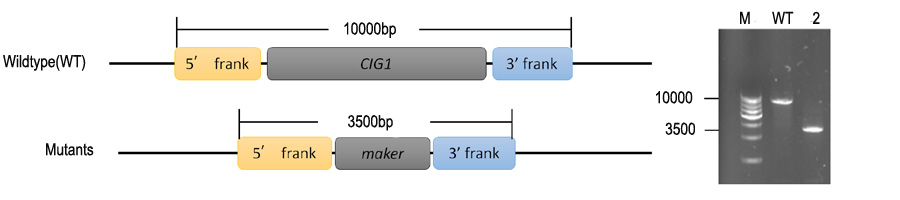


**Fig.S11.** Construction of strains with CIG1 gene knocked out.


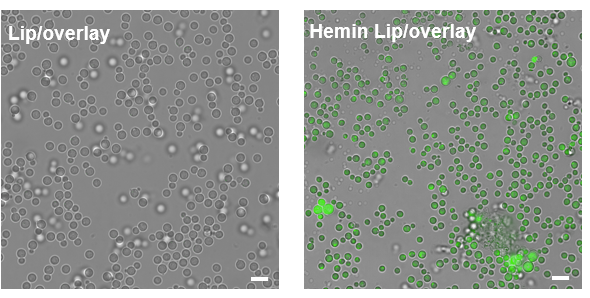


**Fig.S12.** Fluorescence images of Lip and Hemin Lip uptake by

*C. neoformans* H99. (Scale bar, 10 μm).


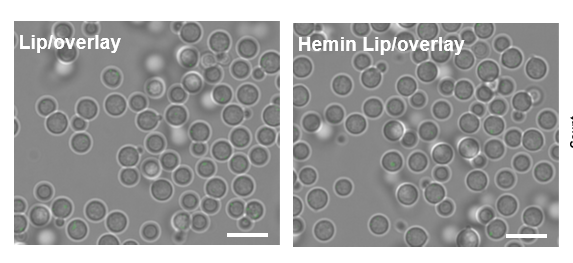


**Fig.S13.** Fluorescence images showing the uptake of Lip and Hemin Lip

by the *cig1*Δ mutant. (Scale bar, 10 μm).


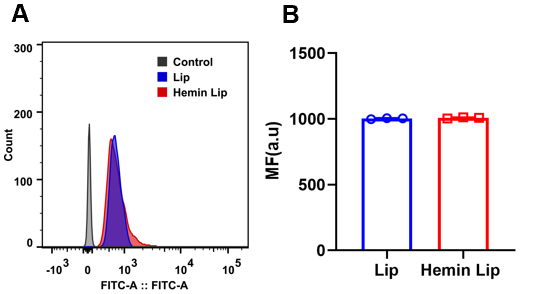


**Fig.S14.** (A) Flow cytometry analysis of Lip and Hemin Lip uptake by the

*cig1*Δ mutant. (B) Mean fluorescence intensity of Coumarin-6 determined by

flow cytometry. Data are presented as mean ± SD (n = 3).

**
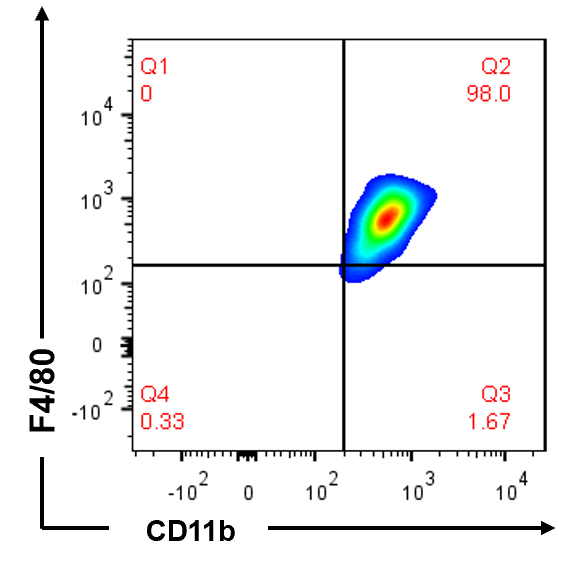
**

**Fig.S15.** Flow cytometry analysis of F4/80 and CD11b expression in

BMDMs.


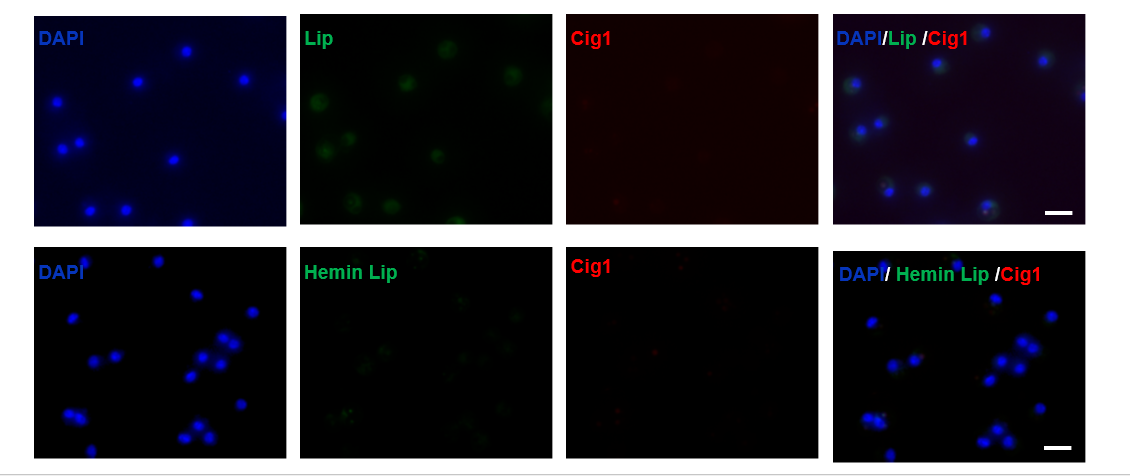


**Fig.S16.** Fluorescence images of *cig1*Δ mutant-infected BMDMs after 2 h

incubation with Coumarin-6-labeled Lip. (Scale bar, 20 μm).


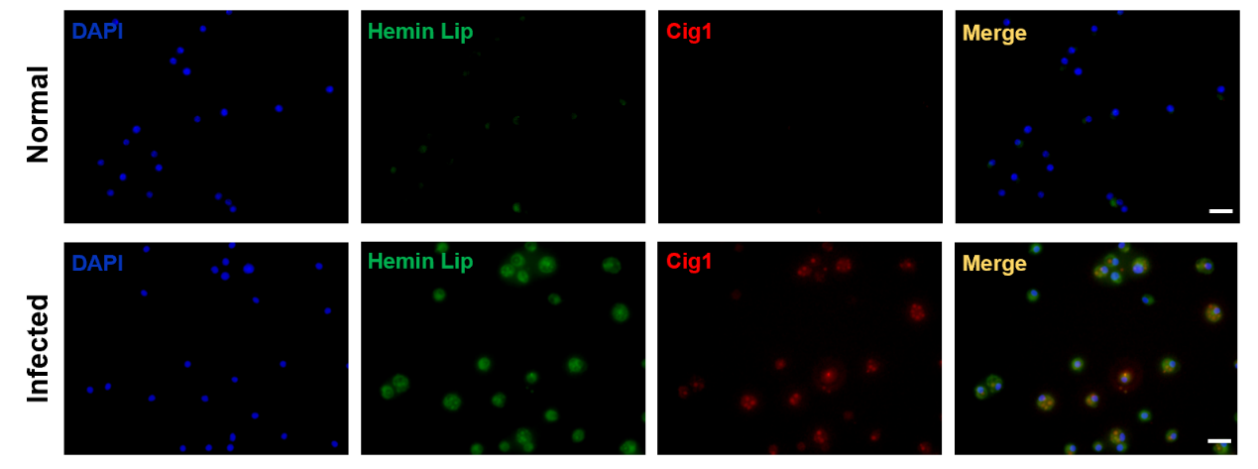
**Fig.S17.** Fluorescence images of normal BMDMs and *C. neoformans* H99-

infected BMDMs after 2 h of incubation with Coumarin-6-labeled Hemin Lip. (Scale bar, 20 μm).

**
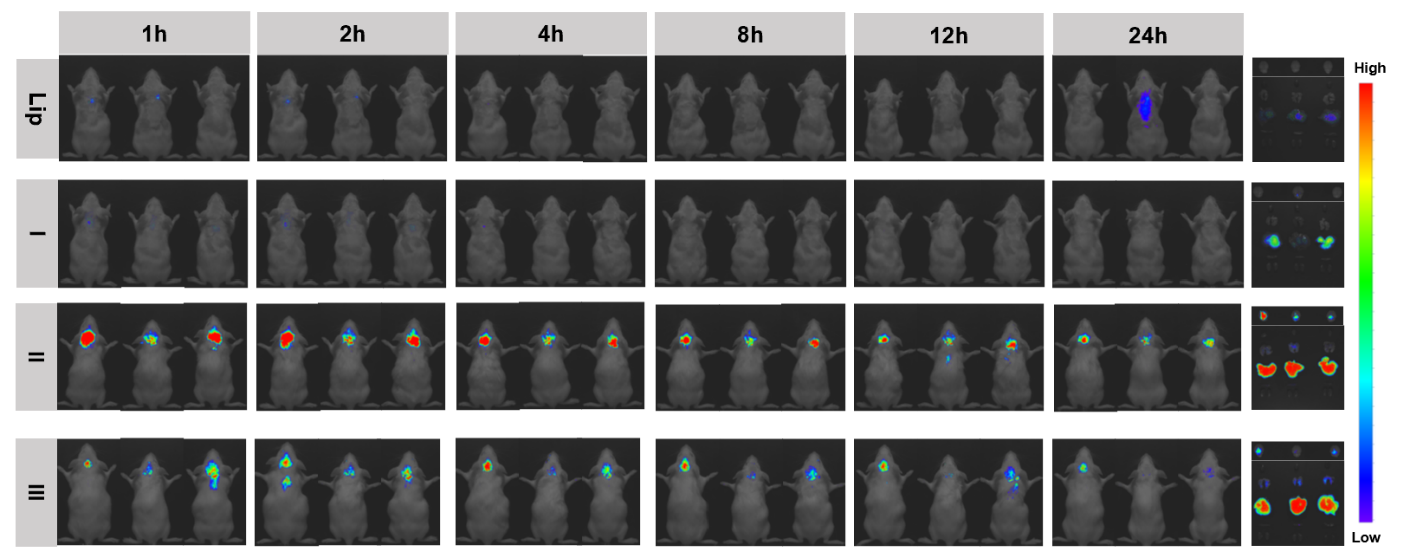
**

**Fig.S18.** *In vivo* and *ex-vivo* images of preparations in normal mice determined by live imaging. Mice used in experiments were [injecte](javascript:;)d with Lip, 1%Hemin Lip (I), 2%Hemin Lip (II) and 3%Hemin Lip (III) respectively through the [tail](javascript:;) [vein](javascript:;). *Ex vivo* images of various tissues from normal mice were collected, mice were sacrificed at 24h after [injection](javascript:;) (n = 3).


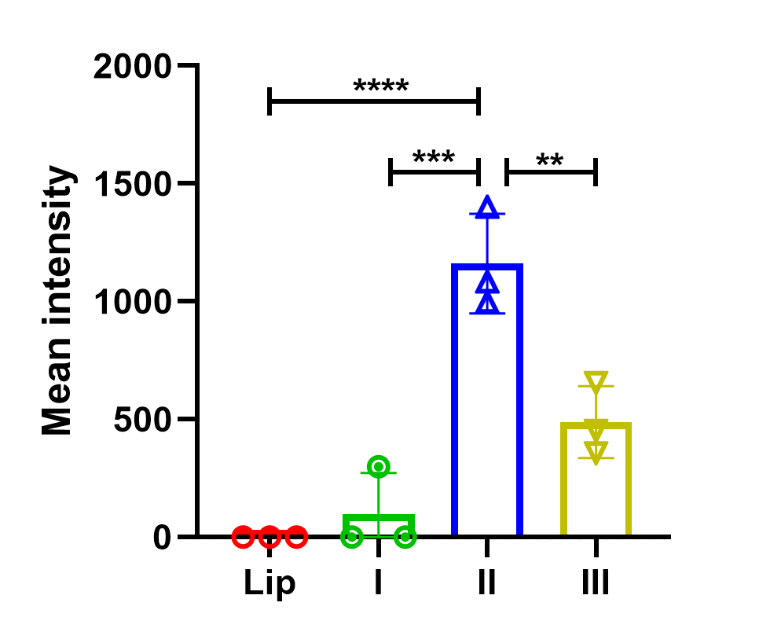


**Fig.S19.** Quantitative *in vivo* imaging analysis of different preparations in normal brains. I :1% Hemin Lip, II :2% Hemin Lip, III :3% Hemin Lip.


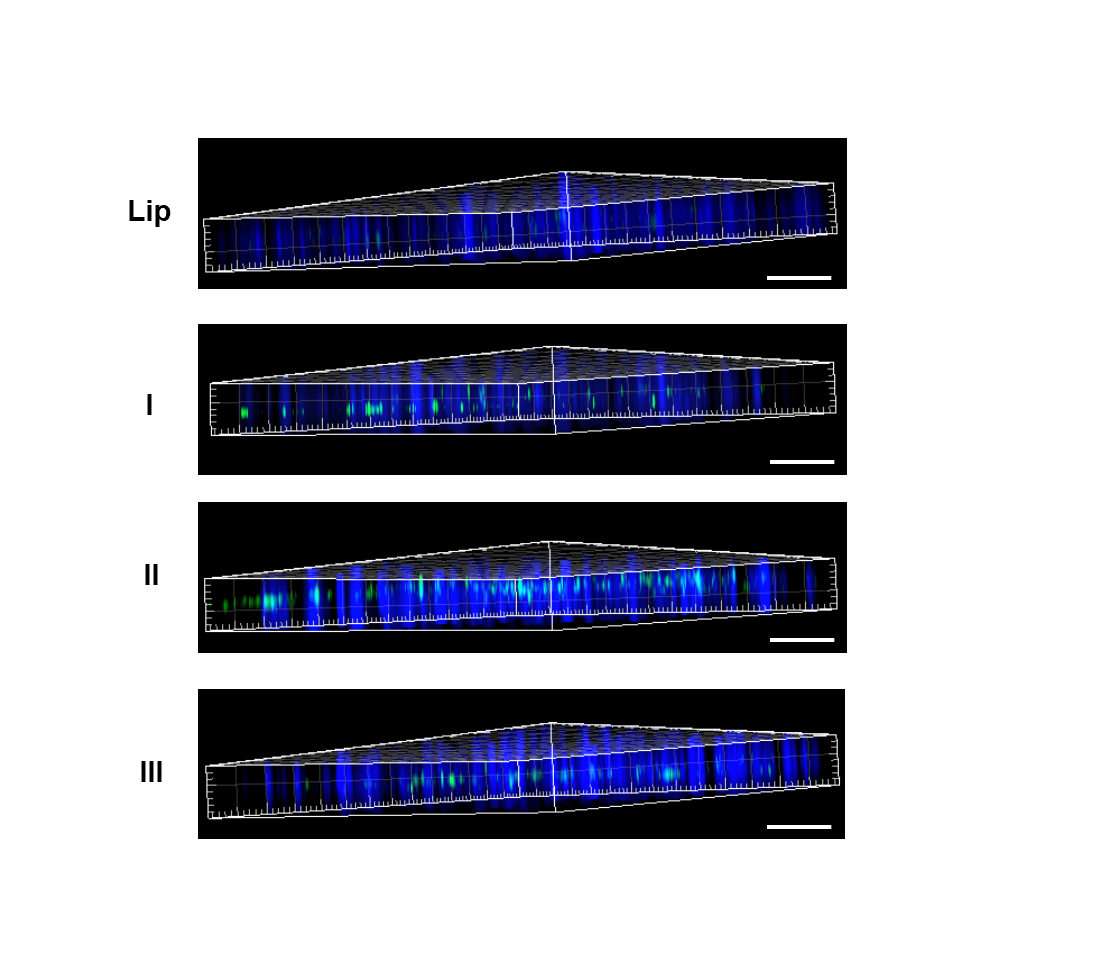


**Fig.S20.** Fluorescence level in 3D images of bEnd.3 cells interacting with Lip, 1% Hemin Lip (I), 2% Hemin Lip (II) and 3% Hemin Lip (III), Lip was labeled with Coumarin-6. (Scale bar, 20 μm).


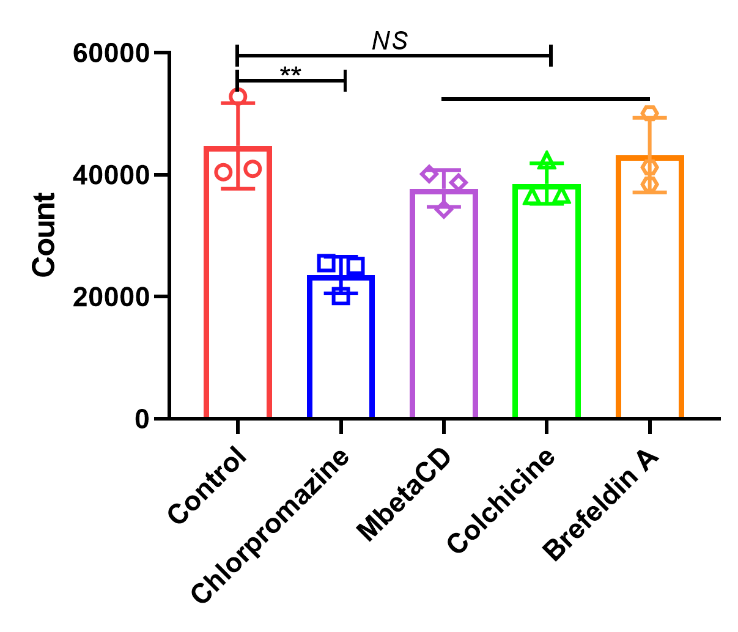


**Fig.S21.** The effect of specific inhibitors targeting distinct endocytic pathways on the transcytosis of Hemin Lip in bEnd.3 cells was assessed using flow cytometry. (n=3)

**Fig.S**
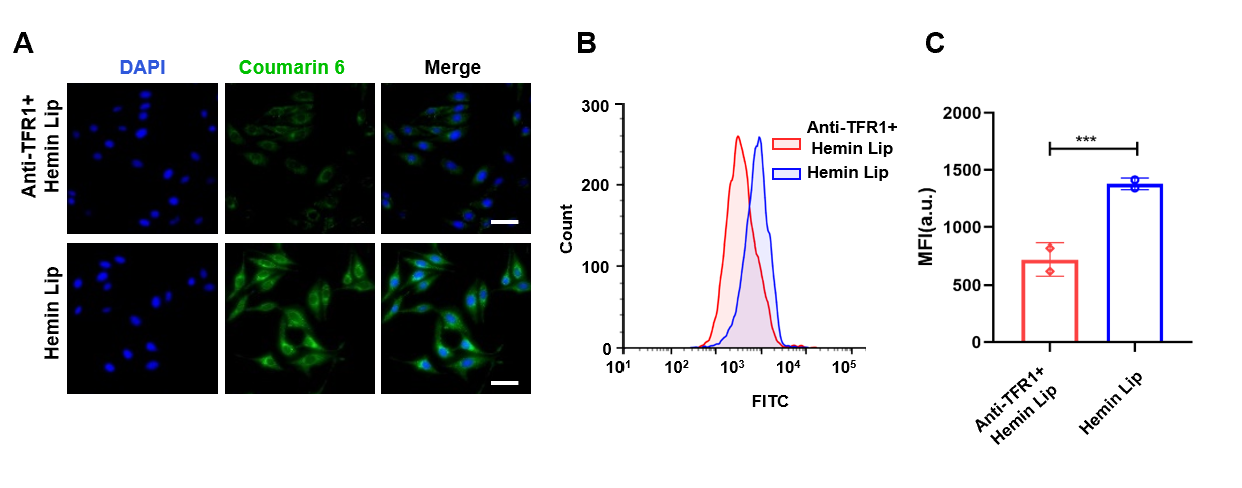
**22.** Following the inhibition of TFR1 using an anti-TFR1 antibody, the cellular uptake of Hemin Lip was assessed through fluorescence microscopy (Scale bar, 50 µm) (A) and flow cytometry (B). (C) The mean fluorescence intensity of Coumarin-6 was quantified via flow cytometry (B). Data are presented as mean ± SD (n = 3).


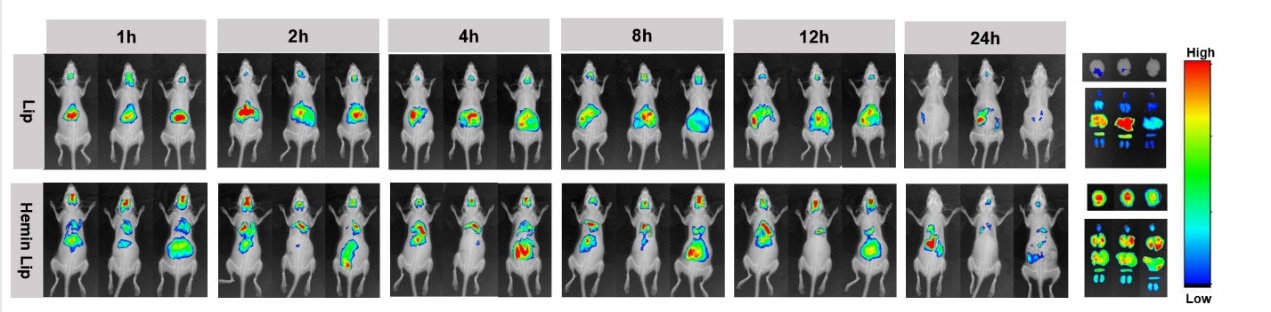


**Fig.S23.** *In vivo* and *ex-vivo* targeting ability of Hemin Lip and Lip in infected mouse models determined by live imaging (n = 3).


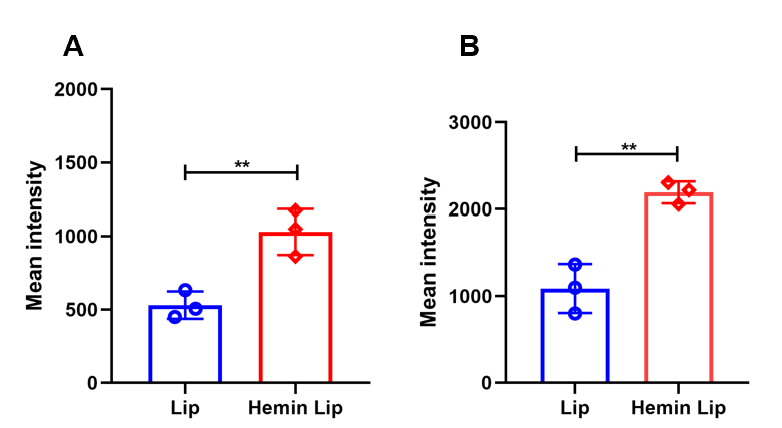


**Fig.S24.** Quantitative *in vivo* imaging analysis of different preparations in infected brains (A) and lungs (B).


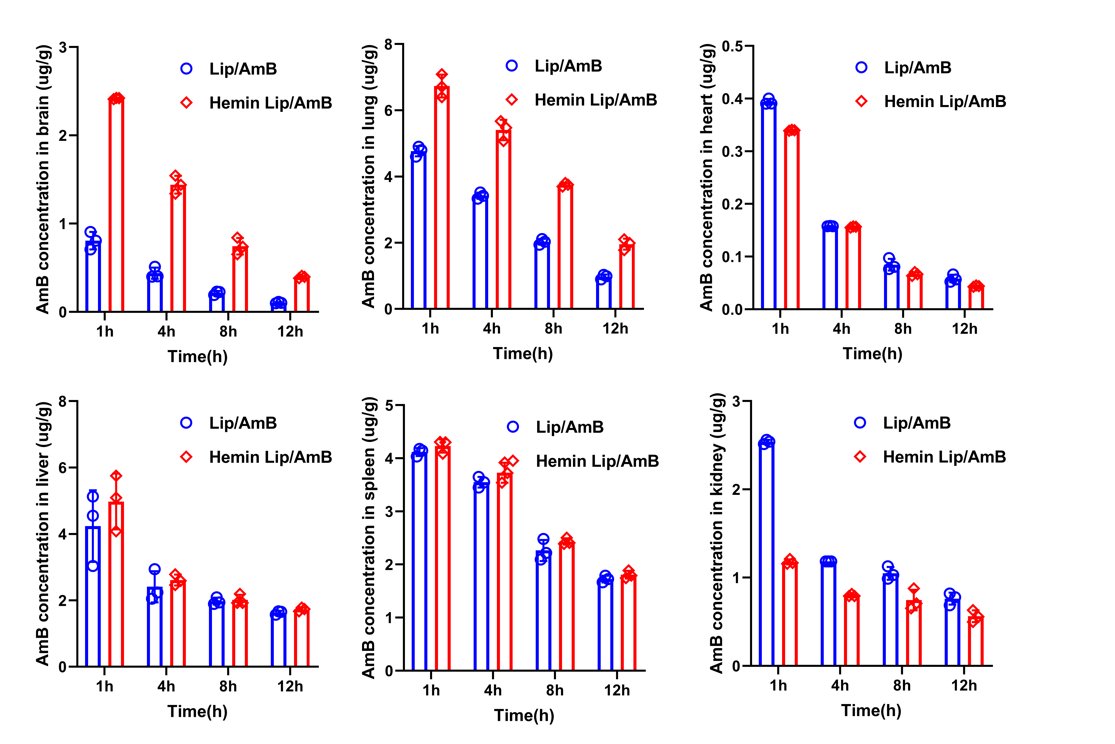


**Fig.S25.** *In vivo* distribution of Lip/AmB and Hemin Lip /AmB in the

brain, lungs, heart, liver, spleen, and kidneys of infected mice at different time points. Data are presented as mean ± SD (n = 3).





**Fig.S26.** Plasma concentration-time curve of AmB in mice after intravenous injection of different AmB loaded preparations for 24 h (the administration dose of AmB was 2 mg/kg). Data are presented as mean ± SD (n = 3).


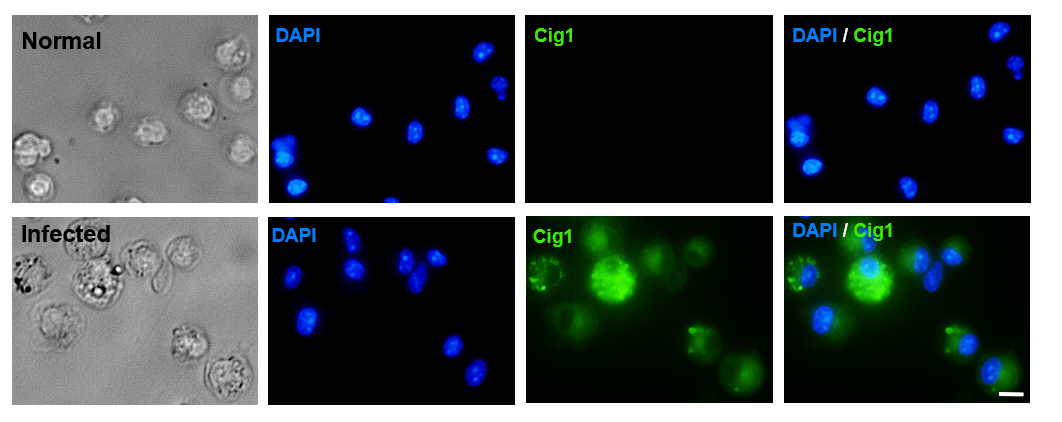
**Fig.S27.** Cig1 protein expression in the pulmonary alveolar macrophages (PAM) of normal mouse and *C. neoformans* H99-infected mouse determined by

immunofluorescence. (Scale bar, 5 μm).

**Fig.S28.** Immunohistochemical analysis of Cig1 protein in the main organs of normal and infected mice
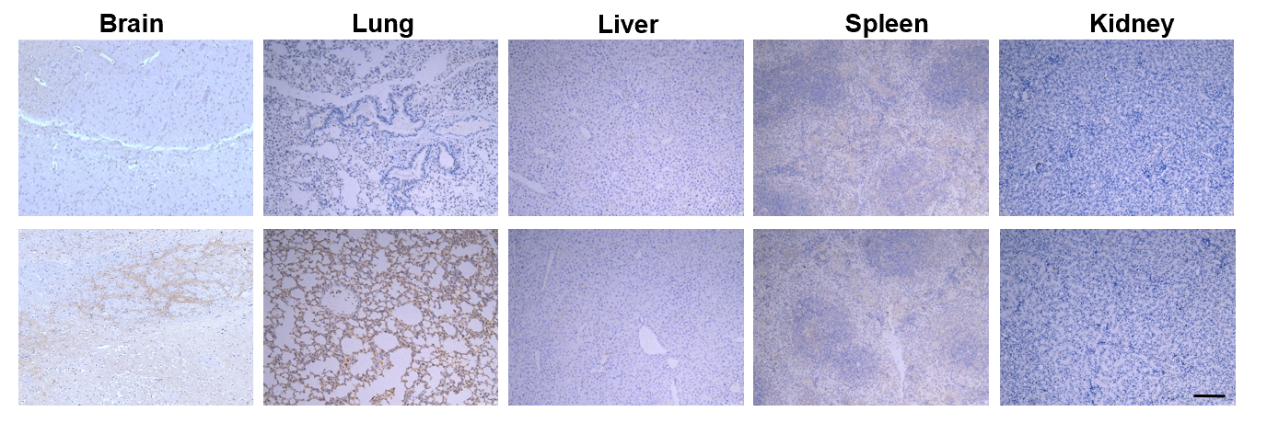
. (Scale bar, 100 μm).


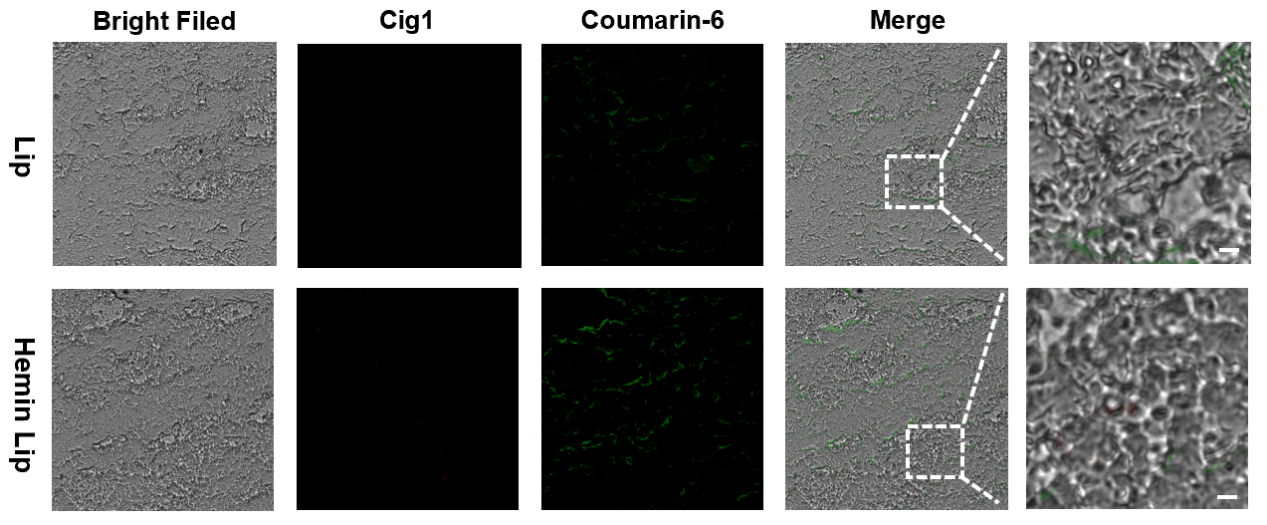


**Fig.S29.** Brain sections from *cig1*Δ mutant infected mice after administration of Hemin Lip and Lip. (Scale bar, 10μm).


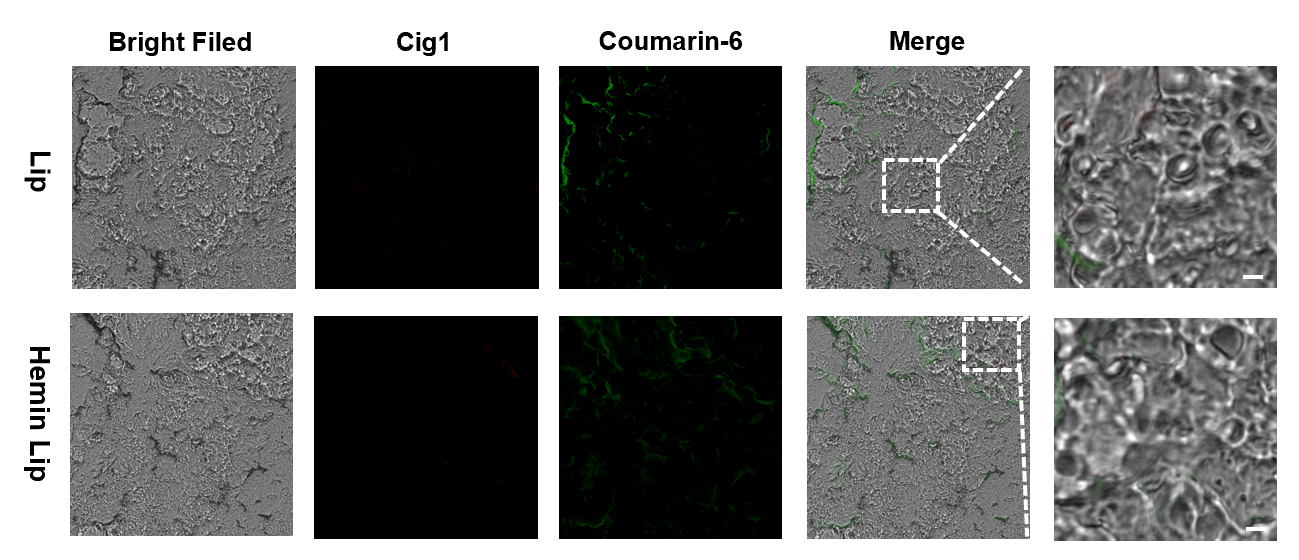


**Fig.S30.** Lung sections from *cig1*Δ mutant infected mice after administration of Hemin Lip and Lip. (Scale bar, 10μnm).


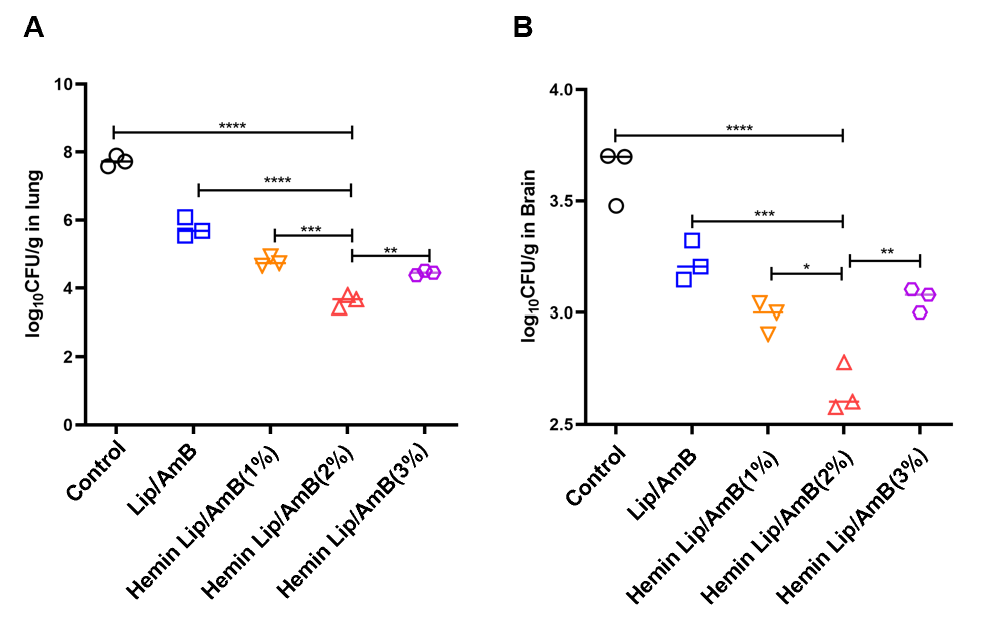


**Fig.S31.** (A) CFU counts in the lungs of infected mice after treatment with different formulations. (B) CFU counts in the brains of infected mice after treatment with different formulations. (n = 3, *p < 0.05, **p < 0.01, ***p < 0.001, ****p < 0.0001).


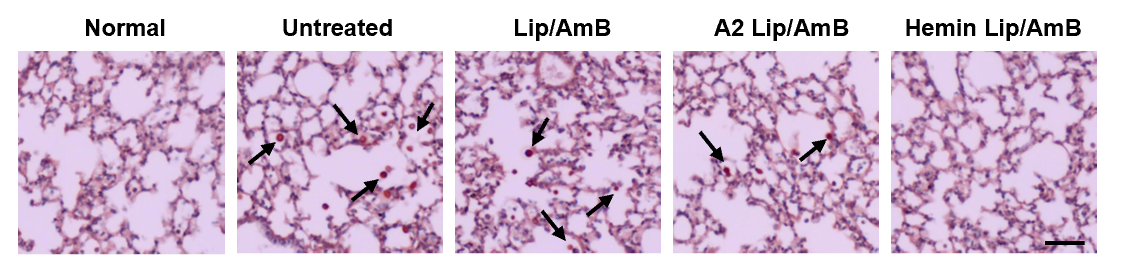


**Fig.S32.** The lung sections of mice after different treatments were stained with PAS and visualized by light microscopy. The *C. neoformans* are indicated by black arrows. (Scale bar, 50 μm).


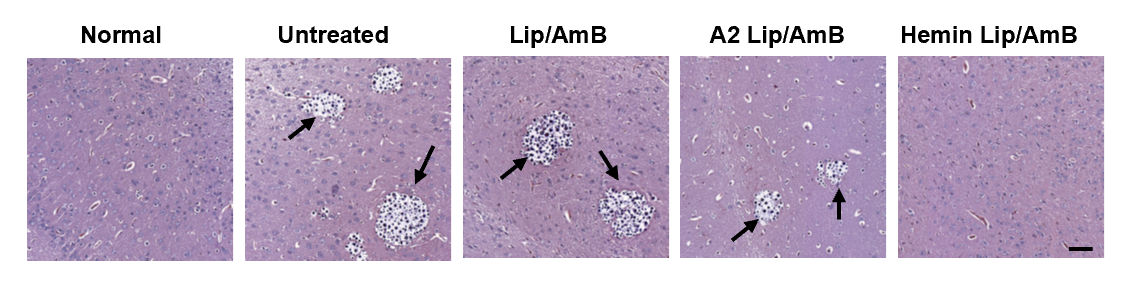


**Fig.S33.** The brain sections of mice after different treatments were stained with GMS and visualized by light microscopy. The *C. neoformans* are indicated by black arrows. (Scale bar, 50 μm).


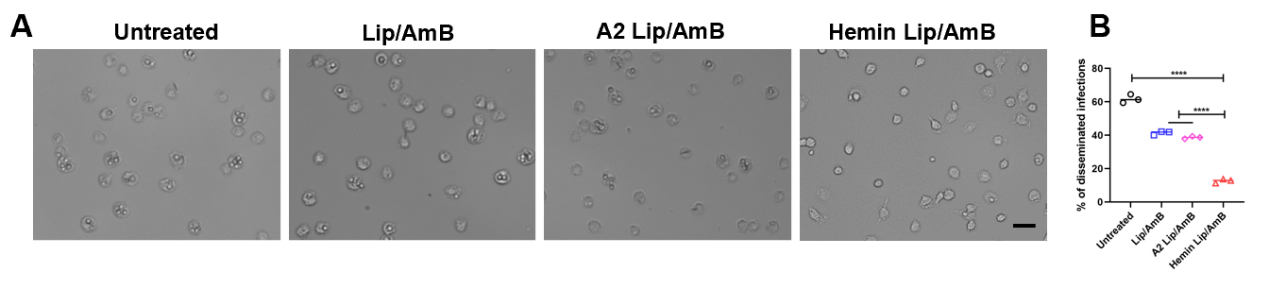


**Fig.S34**. *In vivo* therapeutic effects of Lip/AmB, A2 Lip/AmB, and Hemin Lip/AmB treatments on intracellular *C. neoformans*. (Scale bar, 20 μm).


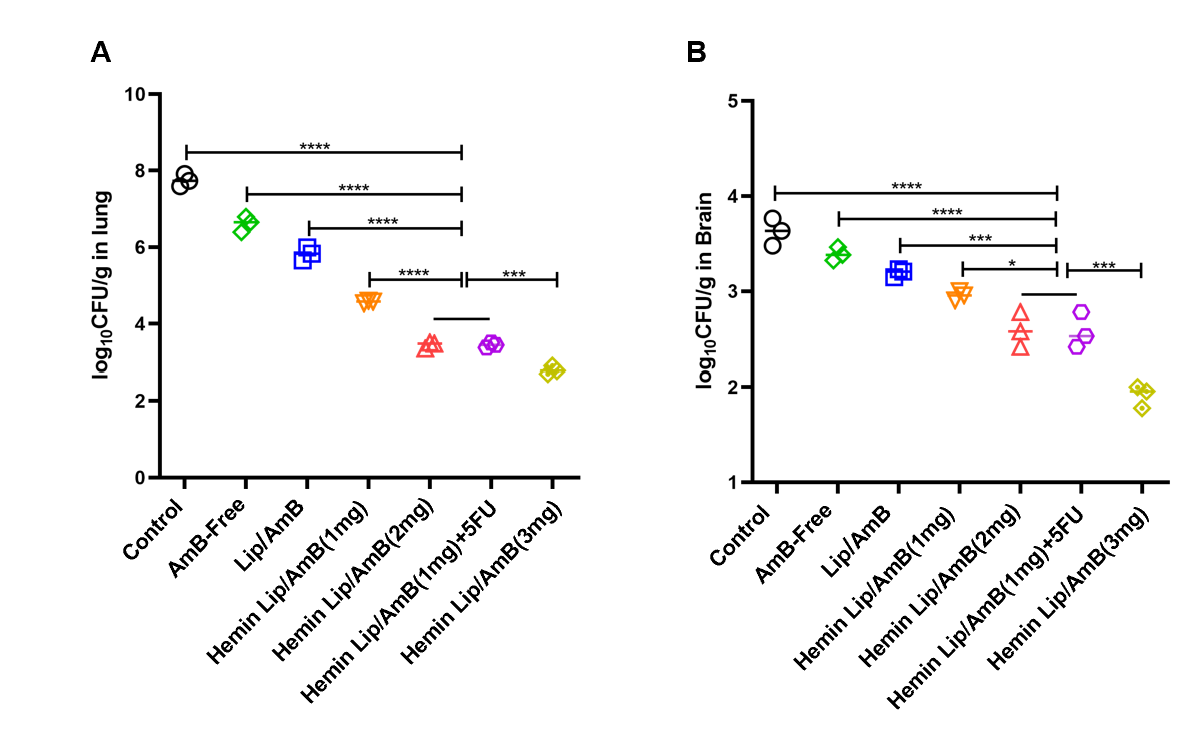
**Fig.S35.** (A) CFU counts in the lungs of infected mice after treatment with different formulations. (B) CFU counts in the brains of infected mice after treatment with different formulations. (n = 3, *p < 0.05, ***p < 0.001, ****p < 0.0001).


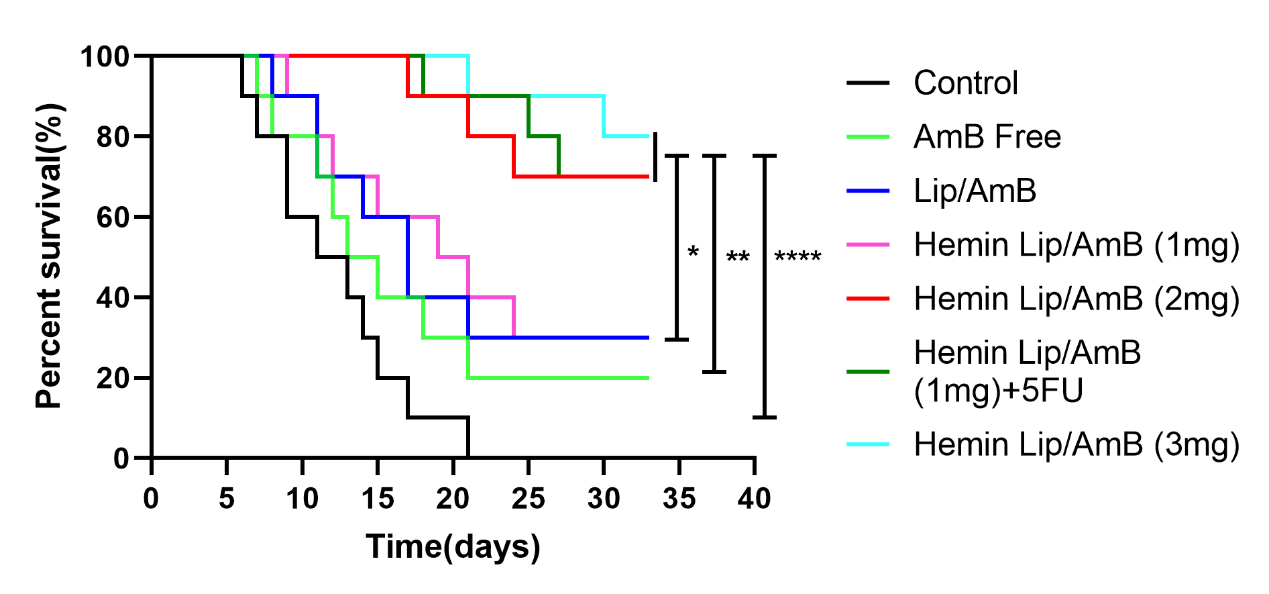


**Fig.S36.** Survival rate of infected mice after treatment with different formulations. (n = 10)


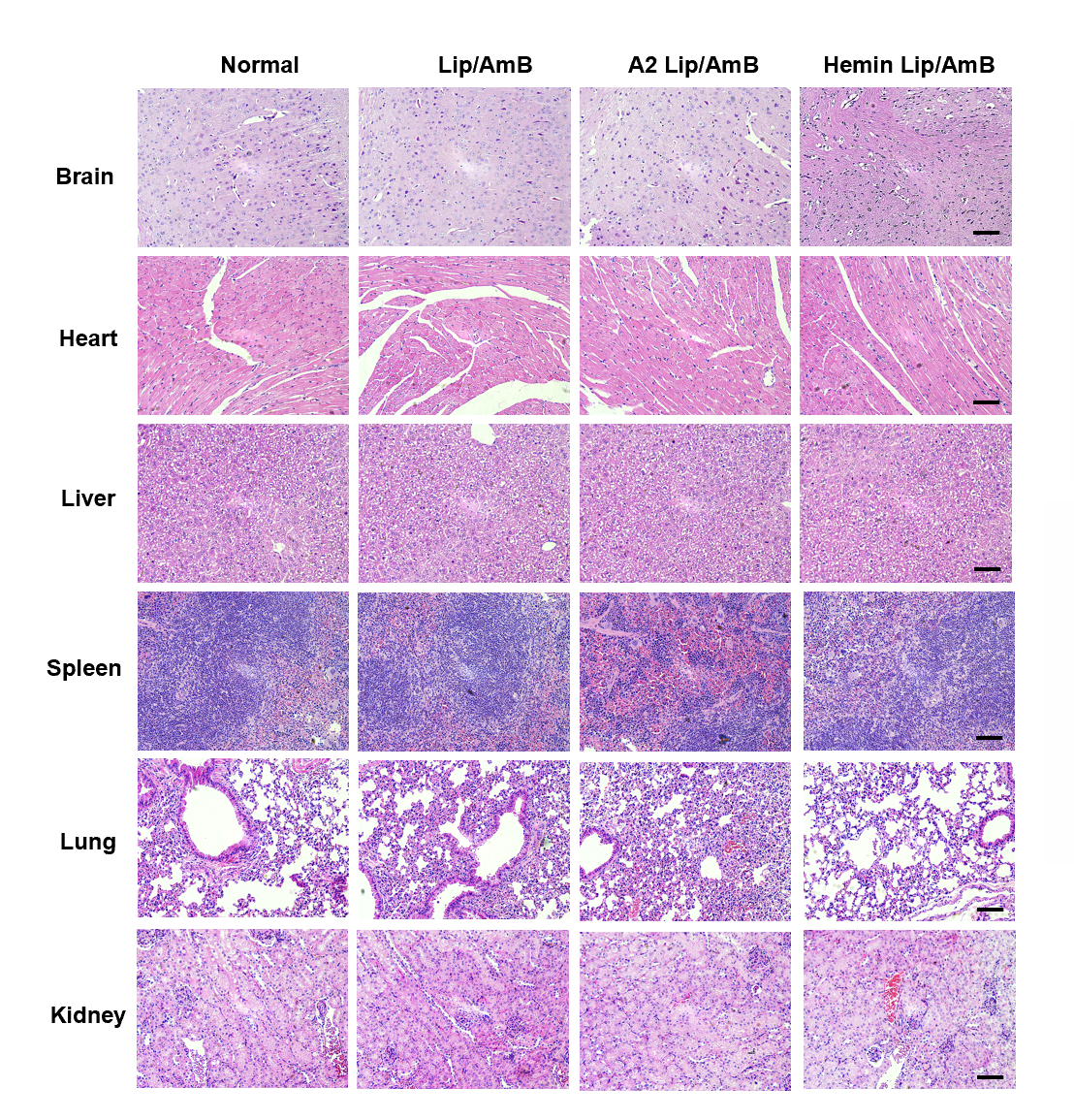


**Fig.S37.** H&E staining of major organs. (Scale bar, 100 μm).


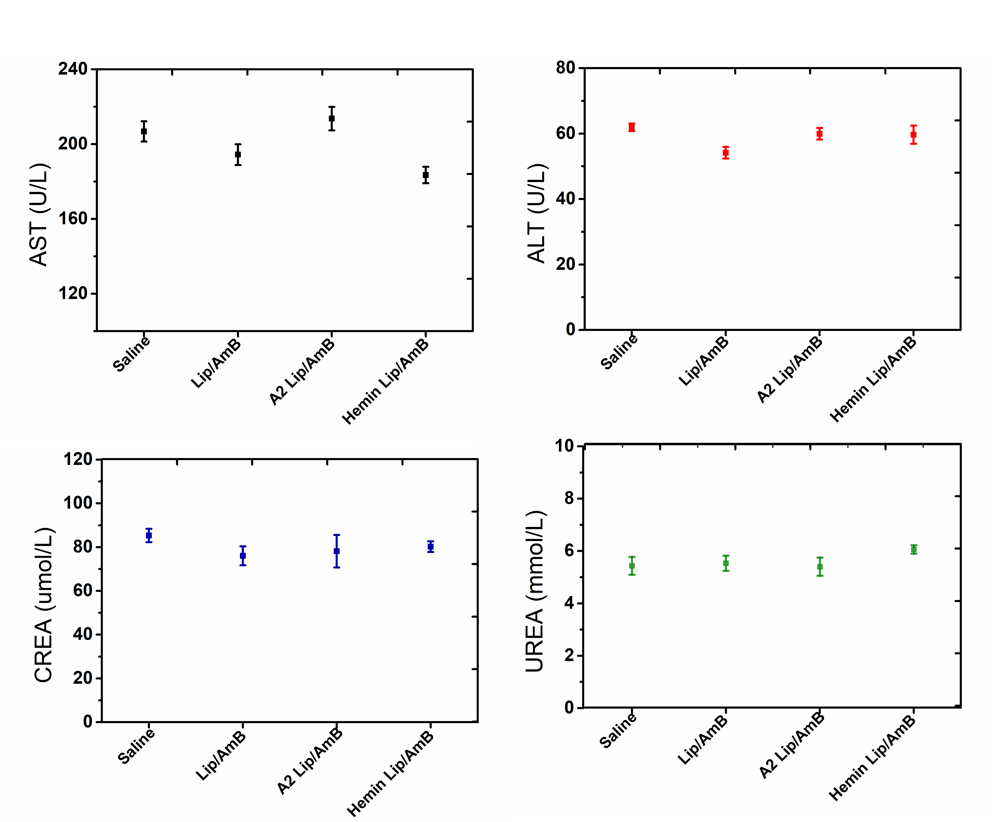


**Fig.S38.** Main physiological indexes of mice after treated with saline, Lip/AmB, A2 Lip/AmB and Hemin Lip/AmB. Data are presented as mean ± SD (n = 3).

**Fig.S**
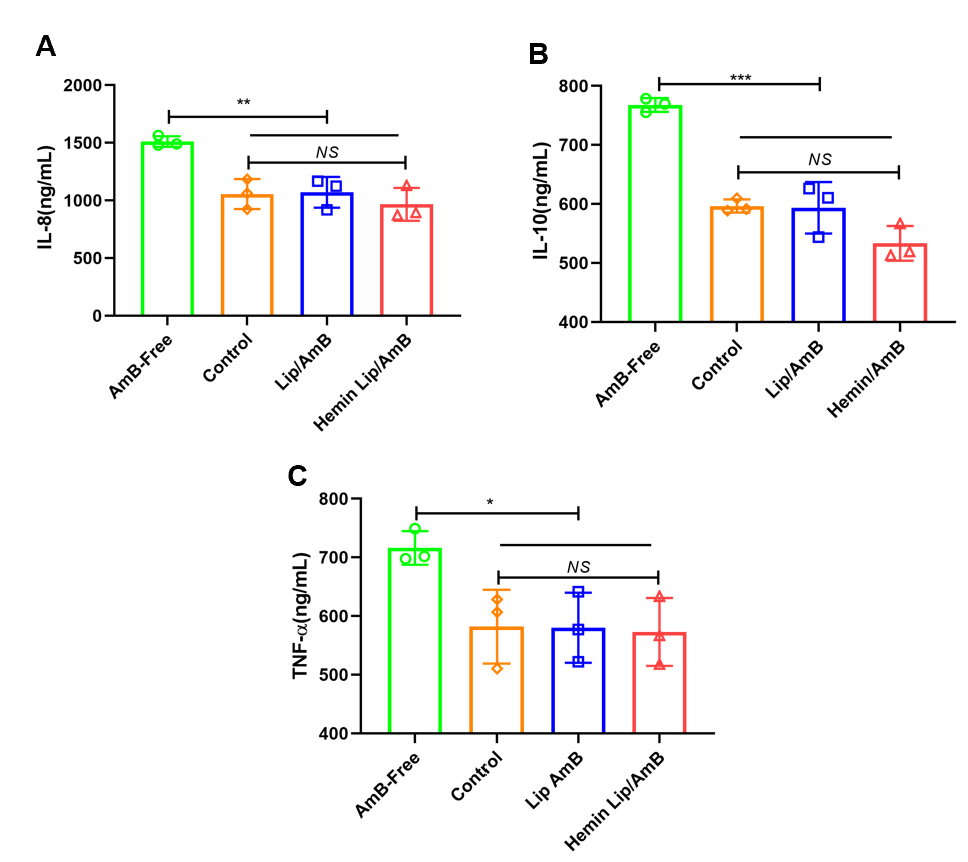
**39.** ELISA analysis of cytokine levels in serum after injection of different liposomes. Data are presented as mean ± SD (n = 3)


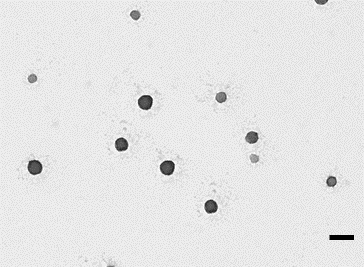


**Fig.S40.** TEM images of BSA NP. (Scale bar, 200 nm).

**
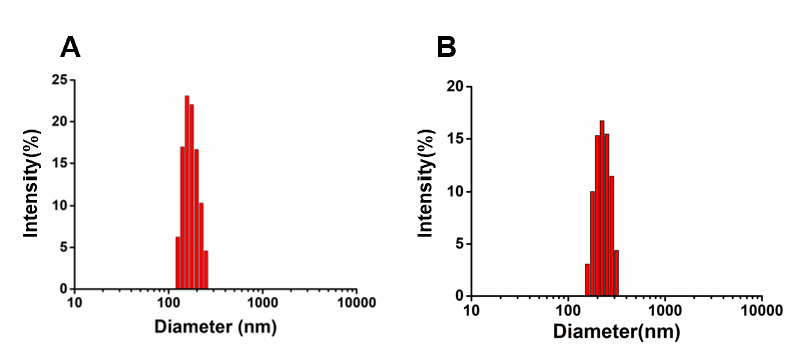
**

**Fig.S41.** Size distribution of BSA NP (A) and Hemin@BSA NP (B).

Table S1. Main pharmacokinetic parameters of AmB formulations in rats.

| Parameters | T_1/2_  (h) | AUC_0-t_  （μg/ml*h） | CL  (mg/kg) /h | Cmax  (μg/mL) |
| --- | --- | --- | --- | --- |
| Lip/AmB | 19.94 | 17.34 | 0.09 | 8.67 |
| Hemin Lip/AmB | 22.44 | 19.39 | 0.09 | 8.76 |

Table S2. Death Times and Symptoms of Mice after Administration Hemin Lip/AmB (n=10 per Treatment Dose)

| Dose (mg/kg) | Female | | Male | | Toxicity responses | |
| --- | --- | --- | --- | --- | --- | --- |
|  | Death/total | Mortality (%) | Death/total | Mortality (%) |  |  |
| 1 | 0/10 | 0 | 0/10 | 0 | - |  |
| 2 | 0/10 | 0 | 0/10 | 0 | - |  |
| 4 | 0/10 | 0 | 0/10 | 0 | Mild toxicity |  |
| 6 | 3/10 | 30 | 2/10 | 20 | Obvious toxicity |  |
| 8 | 7/10 | 70 | 6/10 | 60 | Severe toxicity |  |
| 12 | 10/10 | 100 | 10/10 | 100 | Severe toxicity |  |
